# Binning sequences using very sparse labels within a metagenome

**DOI:** 10.1186/1471-2105-9-215

**Published:** 2008-04-28

**Authors:** Chon-Kit Kenneth Chan, Arthur L Hsu, Saman K Halgamuge, Sen-Lin Tang

**Affiliations:** 1 Dynamic Systems & Control Group, Department of Mechanical Engineering, The University of Melbourne, Australia; 2 Research Centre for Biodiversity, Academia Sinica, Taipei, Taiwan

## Abstract

**Background:**

In metagenomic studies, a process called binning is necessary to assign contigs that belong to multiple species to their respective phylogenetic groups. Most of the current methods of binning, such as BLAST, *k*-mer and PhyloPythia, involve assigning sequence fragments by comparing sequence similarity or sequence composition with already-sequenced genomes that are still far from comprehensive. We propose a semi-supervised seeding method for binning that does not depend on knowledge of completed genomes. Instead, it extracts the flanking sequences of highly conserved 16S rRNA from the metagenome and uses them as seeds (labels) to assign other reads based on their compositional similarity.

**Results:**

The proposed seeding method is implemented on an unsupervised Growing Self-Organising Map (GSOM), and called Seeded GSOM (S-GSOM). We compared it with four well-known semi-supervised learning methods in a preliminary test, separating random-length prokaryotic sequence fragments sampled from the NCBI genome database. We identified the flanking sequences of the highly conserved 16S rRNA as suitable seeds that could be used to group the sequence fragments according to their species. S-GSOM showed superior performance compared to the semi-supervised methods tested. Additionally, S-GSOM may also be used to visually identify some species that do not have seeds.

The proposed method was then applied to simulated metagenomic datasets using two different confidence threshold settings and compared with PhyloPythia, *k*-mer and BLAST. At the reference taxonomic level Order, S-GSOM outperformed all *k*-mer and BLAST results and showed comparable results with PhyloPythia for each of the corresponding confidence settings, where S-GSOM performed better than PhyloPythia in the ≥ 10 reads datasets and comparable in the ≥ 8 kb benchmark tests.

**Conclusion:**

In the task of binning using semi-supervised learning methods, results indicate S-GSOM to be the best of the methods tested. Most importantly, the proposed method does not require knowledge from known genomes and uses only very few labels (one per species is sufficient in most cases), which are extracted from the metagenome itself. These advantages make it a very attractive binning method. S-GSOM outperformed the binning methods that depend on already-sequenced genomes, and compares well to the current most advanced binning method, PhyloPythia.

## Background

With the advancement of technology, genome sequencing projects are moving from the study of single genomes to the examination of genomes in a community. This new study, metagenomics, allows culture-independent and sequence-based studies of microbial communities. A metagenomic project generally starts by using Whole Genome Shotgun (WGS) sequencing [1-6] on environmental samples to acquire sequence reads, followed by assembling sequence reads, gene prediction, functional annotation and metabolic pathway construction. A necessary step in metagenomics, which is not required in single genome sequencing, is called binning. The binning process sorts contigs and scaffolds of multiple species, obtained from WGS sequencing, into phylogenetically related groups (bins). The resolution of phylogenetic grouping can vary from high levels such as domain, down to low levels such as strain of a given microorganism, depending on a number of factors such as the binning method used, community structure and sequencing quality and depth [7]. As the community structure is inherent in the nature of the environmental sample collected, and sequencing quality and depth rely heavily on the sequencing technology and sample size, the major focus of computational techniques is on the method of binning.

A number of binning methods are currently available that fall into two broad categories: sequence similarity-based and sequence composition-based. Sequence similarity-based binning methods, for example BLAST, classify sequences based on the distribution of BLAST hits of predicted genes to taxonomic classes. Composition-based methods discriminate genomes by analysing the intrinsic features of sequence encoding preferences, such as GC content [8], codon usage [9] or oligonucleotide frequencies [10-12], for different genomes. Different approaches to extracting sequence features have been proposed. Some composition-based binning methods include *k*-mer [11], oligonucleotide-frequency-based clustering with Self-Organising Maps (SOM) [13,14], PhyloPythia [15] and TETRA [16,17], all of which have yielded promising results. The *k-*mer method calculates the oligonucleotide frequencies of all sequence fragments and compares them to a reference set of completed genomes. Clustering of oligonucleotide frequencies with SOM can be used in two different ways: directly clustering the metagenomic sample sequences using different oligonucleotide frequencies as features [13], or building an SOM classifier using sequences in existing databases and then assigning metagenomic samples to the closest matching node in the classifier [14]. PhyloPythia follows a similar approach to the SOM classifier method, building a classification model from the sequenced genomes and assigning sample sequences to phylogenetic clades according to the similarity of metagenomic sequence patterns. Two types of PhyloPythia models can be built, a generic model and a sample-specific model. The generic model is constructed using the sequence patterns of a reference set of isolated genomes and the sample-specific model includes additional marker-gene labelled sequence fragments (around 100 kb length) from each of the dominant population of the sample. In contrast, TETRA follows the SOM clustering approach that does not require reference genomes. It bins the species-specific sequences by comparing the pairwise tetranucleotide-derived z-score correlations of every sequence.

The above named binning methods can be divided, from a machine learning perspective, into supervised and unsupervised learning methods. Methods that build a classifier using the knowledge of completed genomes belong to the class of supervised learning methods, such as BLAST, *k-*mer and the SOM classifier approach. However, considering the current amount of known genomes, which is insufficient to represent the almost limitless microbial genomes [18], the resultant under-representation of training samples can be detrimental to these supervised-learning methods. PhyloPythia, being the latest of the binning methods addressed here, has been shown to be able to classify sequence reads with great accuracy when training data is highly relevant to the species being assigned, which can either be from a set of reference genomes or using 100 kb length marker-gene labelled sequences, which may not always exist to build a sample-specific model. Unsupervised-learning methods do not have this dependence on training data. TETRA falls into this category, but it focuses on the long fosmid-sized 40 kb fragments and its all-verse-all pairwise comparison matrix can quickly become intractable for a large number of sequences. The direct clustering approach with SOM was tested to separate 1 kb and 10 kb sequence fragments derived from 65 bacteria and 6 eukaryotes, but only the 10 kb tests showed clear species-specific separations [13]. Nevertheless, without the reference sequences, the results lack identifiable labels to resolve for the clusters of sequence reads produced.

To circumvent dependence on the knowledge of completed genomes and provide meaningful clustering results, we propose a semi-supervised seeding algorithm that uses very small amounts of labelled samples, extracted from metagenomes, for binning metagenomic sequences. The seeding method is a post-processing method that can be applied to an unsupervised clustering algorithm of choice, for example, SOM, Growing Self-Organising Maps (GSOM) [19], or the Incremental Grid Growing Neural Network [20], which also provide dimensionality reduction for data visualisation. Comparing the clustering qualities of SOM and GSOM indicated that GSOM has a better clustering quality and faster computational speed, as confirmed by experiments conducted on other datasets [21,22].

Therefore, in this paper, we implemented the proposed seeding method on GSOM, henceforth abbreviated as S-GSOM. The performance of S-GSOM for binning metagenomic sequences is demonstrated by applying it to simulated metagenomic datasets. Preliminary tests were first conducted to compare S-GSOM with the following well-known semi-supervised learning methods: Constraint-Partitioning K-Means (COP K-Means), Constrained K-Means, Seeded K-Means and the Transductive Support Vector Machine (TSVM) on the task of separating sequence fragments created from 10, 20 and 40 prokaryotic species randomly sampled from the NCBI genome database. For a comparison with current binning methods, further tests of S-GSOM were performed using the recently published simulated metagenomic datasets that were created to evaluate the fidelity of metagenomic processing methods [7].

## Methods

### Datasets preparation and data pre-processing for separation of prokaryotic DNA sequences

#### Generation of independent simulated metagenomic data

The most updated NCBI database contains 488 completed Archaea/Bacteria genomes [23]. Genome sequences of 10, 20 and 40 species were randomly selected from the NCBI database. In total, seven random sets were independently drawn from the database, replacing them each time. Three sets were drawn for each of the 10 and 20 species datasets and only one set for the 40 species dataset, considering the limitations imposed by the available computing resources. (For computational time estimation without the seeding procedure, please refer to our previous publication [22]). For convenience, we abbreviated the datasets created in the form of '*X*Sp-Set*Y*' where '*X*' denotes number of species and '*Y*' represents the number in the draw. For example, the first dataset containing 10 random species is denoted by the abbreviation '10Sp-Set1' and the other datasets are named in the same manner. The lists of species in all datasets can be found in supplementary material [see Additional file 1].

**Figure 1:**
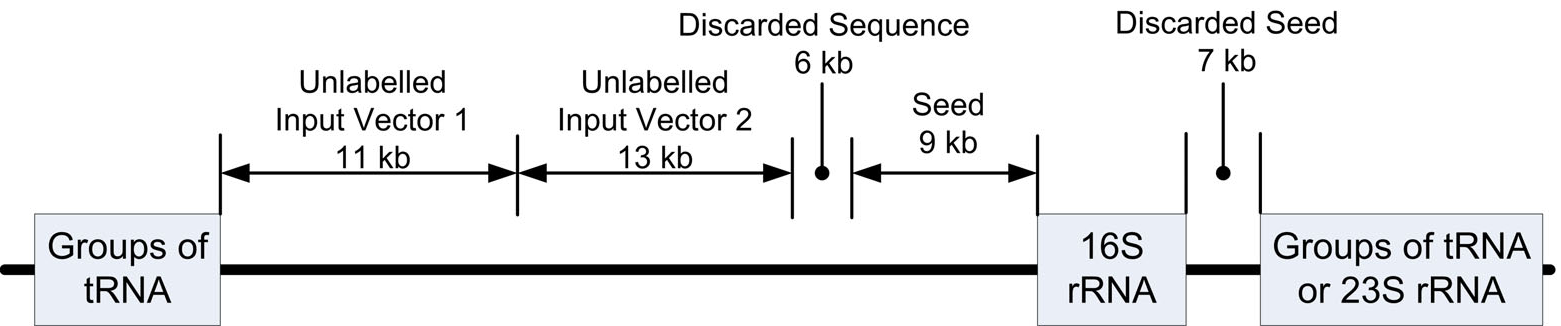
**An example for preparing unlabelled input vectors and seeds**. Unlabelled input vectors and seeds are prepared by avoiding the RNA sequences.

The first step in pre-processing involves the extraction of fragments from the collected datasets. Since rRNA and tRNA sequences are very similar among different species [24], they are likely to interfere with the clustering process when nucleotide frequencies are used as training features. Therefore, we take the precaution to reduce noise by avoiding the inclusion of these RNA sequences. This noise reduction is also valid in practical situations because all these RNA sequences can be easily identified based on sequence similarity against public databases, e.g. RDP [25] or GenBank [23], and are excluded from the clustering process. Figure 1 contains an example for preparing unlabelled input vectors and seeds for clustering algorithms, and a typical extraction process is as follows:

1. Identify all rRNA and tRNA sequences in the genome.

2. Obtain 8–13 kb length seeding sequences that flank the 16S rRNA sequence (see later for more details on seeding sequences). Ensure that a seeding sequence does not overlap other rRNA and tRNA sequences.

3. Remove all rRNA, tRNA and seeding sequences from the genome to obtain initial sequence fragments.

4. Divide initial sequence fragments into non-overlapping 8–13 kb (determined randomly) length fragments. Fragments shorter than 8 kb are discarded.

5. Nucleotide frequencies of each sequence fragments are computed using a sliding window of the size of 4 bases (tetranucleotide frequencies). A total of 256 (4^4^) combinations of nucleotide usages are represented in the vector form of 256 dimensions.

6. Since the sequences were cut into random lengths between 8 kb and 13 kb fragments, the tetranucleotide frequencies also need to be normalised relative to the length of the fragments (i.e. frequency per base).

The choice to use 8 kb as the minimum sequence length is because the number of chimeric sequences becomes significant for sequence length < 8 kb using the currently available assemblers [7], and the chimeric sequences will reduce the binning accuracy leading to meaningless results. The same reason explains the use of ≥ 8 kb sequence length for the simulated metagenomic datasets created in Mavromatis et al. [7]. The restriction of having seed length between 8 kb and 13 kb is applied to provide a standardised rule for all sequences, whether seed sequence or not, in the generation of the artificial datasets. The choice to use tetranucleotide frequency follows that of the work of Abe et al. [13,14], who tested di-, tri- and tetranucleotide frequencies as training features and found that tetranucleotide frequencies have a better species separation among the three oligonucleotide frequencies. In addition, Sandberg et al. [11] showed that a longer nucleotide sequence represents the genome more specifically and Teeling et al. [16] mentioned that the correlations of tetranucleotide usage patterns is high between intragenomic fragments and low between intergenomic fragments. Therefore, the tetranucleotide frequency was used as the training feature for the clustering algorithms.

#### Justification and identification of seeds

Prior to applying the semi-supervised learning methods for binning of metagenomic sequences, small amounts of labelled data or seeds are to be identified from the datasets of sequence fragments for training the classifiers. However, exact taxons are only known for experimentally identified species, which are still few in number, making them impractical for metagenomic applications. Additionally, the labelled data should not depend on the existing completed genomes and should be informative enough to describe the species. In this work, we employed the flanking sequences of conserved 16S rRNA as seeds for the semi-supervised clustering algorithm. The flanking sequences of highly conserved 16S rRNA sequences can be easily obtained from sequencing results or by Southern blot hybridisation in the WGS genomic DNA library and sequencing the positive clones. These flanking sequences are often similar within, and only within, the closely related species in terms of nucleotide frequency. This property allows the discrimination of species at a medium resolution level, although it is insufficient for binning strains of the same species.

It is well known that 16S rRNA sequences are highly conserved over the evolution of organisms, such that the difference between various species can be identified from the small number of base changes in the 16S rRNA sequences. Some papers have exploited these small base changes to estimate the number of species in their environmental sequencing samples [2,26,27]. Due to the highly conserved nature of 16S rRNA sequences, that have highly similar nucleotide frequencies even across different species, they cannot be directly used to distinguish species by clustering that uses nucleotide frequency as the training feature. We verified this by clustering sequence fragments with the inclusion of 16S rRNA fragments, and all 16S rRNA sequences tended to group together to form a separate cluster (data not shown). However, the flanking sequences of the 16S rRNA sequences, which can be generated by sequencing with universal primers, do possess enough variations in nucleotide frequency to be used to identify different species, even for new species. Hence, the flanking sequences of the 16S rRNA sequences are excellent candidates for seeds. Nevertheless, due to the nature of pre-processing for obtaining sequence fragments, if there are other rRNA and tRNA sequences in a genome within the length of the sequence fragment on either end of a 16S rRNA sequence, the seeding sequences will be discarded and the genome becomes unseeded. Figure 1 shows one case where the right hand-side seed is discarded because the length of that flanking sequence is shorter than the pre-determined length (randomly chosen between 8 and 13 kb). The 9 kb length of the left-hand-side seed was also determined by a random generator. On the other hand, all seeds in the benchmark datasets were generated using a length of 10 kb.

#### Self-organising maps and growing self-organising maps

The Self-Organising Map (SOM) [28,29] was originally developed to model the cortexes of more developed animal brains. Subsequently, it became a very popular technique in the field of data mining. It is an unsupervised clustering algorithm which can visualise unlabelled high dimensional feature vectors into groups on a lattice grid map. This is achieved by projecting high dimensional data onto a one-, two- or three-dimensional feature map that has a pre-defined regular lattice structure. In the map, every lattice point represents a node whose weight vector has the same dimension as the input vectors. The mapping preserves the data topology, so that similar samples can be found close to each other in the grid map. The grouping of samples can be visualised by comparing the distance between nodes or simply the inputs that have been projected onto the same node. SOM uses a static lattice structure where the size and shape of the lattice is defined before training and remains unchanged throughout training. The SOM algorithm separates training into three phases: initialisation of the map, ordering and fine tuning. The initialisation method of node weight vectors and choice of number of nodes in the map can be crucial to achieve a good quality clustering result for SOM. Generally, the principle components analysis (PCA) is used for initialising the SOM [30] by positioning a fully unfolded map on the plane formed by the first two principle vectors in the input space. The number of nodes, which represents the resolution of the map, needs to be determined by the user. In the ordering and fine tuning phases, each input is presented to the map and a 'winning' node, which has the smallest Euclidean distance to the presented input, is identified. The weight vector of the winning node and its neighbouring nodes are updated by

(1) *w*(*t *+1) = *w*(*t*) + *α *× *h *× [*x*(*k*) - *w*(*t*)].

where *w *is the weight vector of the node, *x *is the input vector (*w*, *x *∈ ℜ^*D *^where *D *is the dimension), *k *is the index of the current input vector, *α *is the learning rate and *h *is the neighbourhood kernel function.

The Growing Self-Organising Map (GSOM) [19,31] is an extension of SOM. It is a dynamic SOM, which overcomes SOM's weakness of a static map structure. As with SOM, GSOM is used for clustering high dimensional data and employs the same weight adaptation and neighbourhood kernel learning as SOM, but has a global parameter of growth named Growth Threshold (*GT*) that controls the size of the map.

The Growth Threshold is defined as

(2) *GT *= - *D *× ln(*SF*).

where *D *is the dimensionality of data and *SF *is the user-defined Spread Factor that takes values [0,1], with 0 representing minimum and 1 representing maximum growth.

There are four phases of GSOM training: initialisation of the map, a growing phase and two smoothing phases. In the initialisation phase, the GSOM is initialised with a minimum single 'lattice grid', depending on whether the rectangular or hexagonal network topology is used. For GSOM that uses a rectangular topology, the minimum single lattice grid consists of four nodes that are connected as a rectangle; when using the hexagonal topology initialisation, there are seven nodes that form a hexagon with an additional node in the centre that is linked to all six other nodes. More details of initial lattice are described in [19,30]. During the growing phase, every node keeps an accumulated error counter and the counter of the winning node (*E*_*winner*_) is updated by

(3) *E*_*winner *_(*t *+1) = *E*_*winner *_(*t*) + ||*x*(*k*) - *w*_*winner *_(*t*)||

If the winning node is at the boundary of the current map and *E*_*winner *_exceeds *GT*, new nodes will be added to the surrounding vacant slots of the winning node. Weight vectors of the new nodes are created by interpolating or extrapolating the weight vectors of existing nodes around the winning node. In the case when *E*_*winner *_exceeds *GT *and the winning node is not a boundary node, *E*_*winner *_is evenly distributed outwards to its neighbouring nodes. The two smoothing phases are for finetuning the weights of nodes and no new node will be added to the map. In this paper, the 2-D hexagonal lattice was used for GSOM, since the hexagonal lattice yields better data topology preservation [32].

#### Seeded growing self-organising map algorithm

It is sometimes difficult to visually identify clusters in a trained GSOM. Previously, we developed a region identification algorithm to automatically identify the clusters based on the distance map which visualises the distance of the weight of node to each of its neighbour nodes [33]. However, in binning, sequence fragments of closely related species will most likely have homologous sequences present in between the clusters that occlude the cluster boundaries in the distance map and cause the region identification algorithm to fail to identify the correct clusters. Therefore, a method is needed to identify clusters in GSOM to make the clustering approach of GSOM practical for binning.

**Figure 2:**
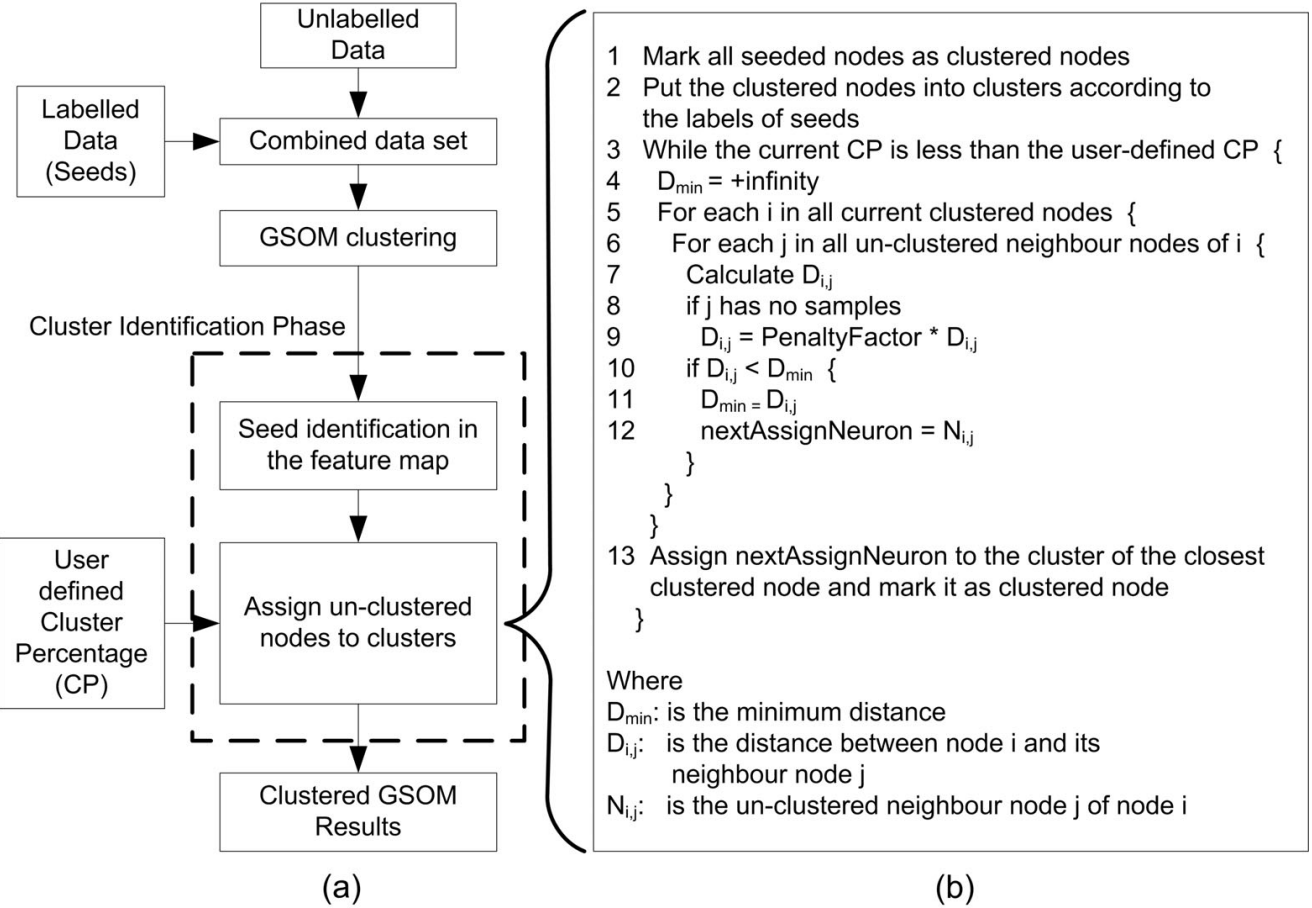
**The S-GSOM algorithm**. (a) Schematic diagram of the clustering process of S-GSOM; (b) The pseudo code for node assigning process in S-GSOM.

The core concept of Seeded GSOM (S-GSOM) is to automatically identify clusters in the feature map using the already-available labelled samples (seeds). The algorithm consists of three core procedures. Firstly, the very small amounts of available or selected seeds (labelled input vectors) are combined with other unlabelled samples (unlabelled input vectors). Secondly, the combined samples (input vectors) are presented to GSOM for training in which the seeds are treated the same as the unlabelled data. Finally, after the normal phases of GSOM training, S-GSOM performs an extra phase, the cluster identification phase, as post-processing. This phase identifies clusters based on the locations of seeds in the trained GSOM and the specified amount of nodes to be clustered. The flowchart of the overall S-GSOM algorithm is given in Figure 2a.

In the cluster identification phase, the seeded nodes, which are nodes that contain the seeds, are identified in the GSOM. An assigning process will then assign the un-clustered nodes (nodes that have not been assigned to a cluster) to clusters. Figure 2b provides the pseudo-code of this assigning process. The number of nodes that will be assigned to a cluster is specified by the Clustering Percentage (CP), *which is the percentage of the number of clustered nodes to the total number of nodes*. The assigning process first creates the initial set of clusters with only the seeded nodes (steps 1 and 2 of pseudo code). The process then iteratively assigns the un-clustered nodes, one by one, to clusters. In each iteration, a set of un-clustered nodes that is adjacent to the clustered nodes is identified. The node within the set that is the shortest Euclidean distance from its neighbouring clustered node will be assigned to the cluster of that clustered node (steps 3 to 13 of pseudo code). However, nodes that do not contain any sample can exist, and these empty nodes most likely represent the boundary of clusters. Therefore, a penalty factor greater than one is multiplied to the actual distance when calculating the distance from an empty node to clustered nodes (step 9 of pseudo code). This will force the algorithm to avoid clustering empty nodes, thus favourably completing the assignment of its own cluster before jumping into other clusters. It is empirically observed that clustering results are not very sensitive to the penalty factor between values of 2 and 5, and a penalty factor value of 2 was used in all our experiments.

**Figure 3:**
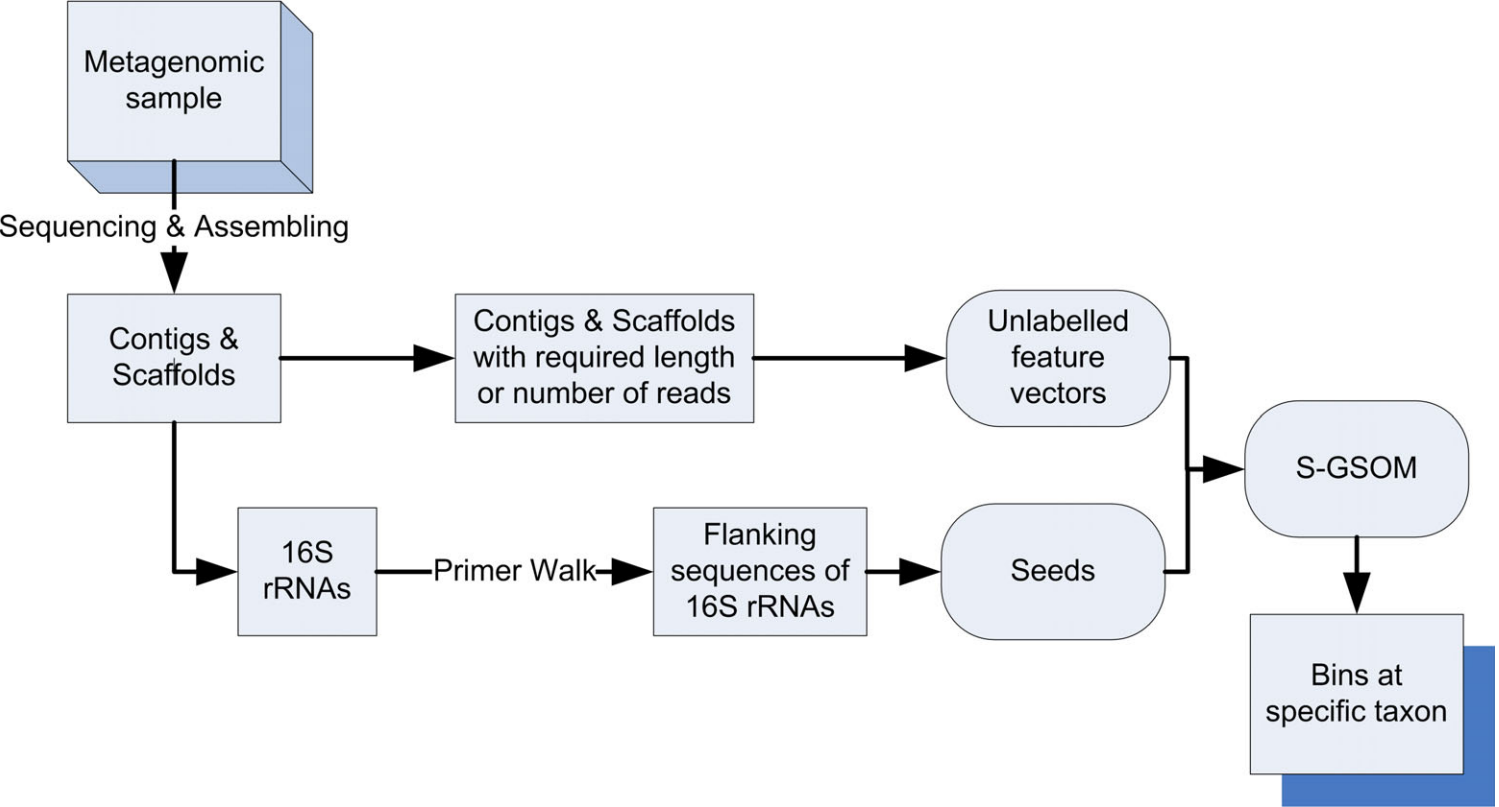
An overview of binning process using S-GSOM.

To illustrate the role of S-GSOM in binning, Figure 3 shows the schematic diagram that explains how S-GSOM fits into the whole binning process. In the binning process, a bin is created for all seeds that are obtained from one 16S rRNA that is the phylogenetic marker of a taxon. A bin may contain multiple seeded nodes that contain the seed sequences. In the process of assigning labels to unlabelled nodes, the taxon label of the seeded node needs to be determined. It is straightforward when there are only seeds that come from the same taxon. However, when seeds in a node come from different taxa, the node will have the label of the taxa which the majority of seeds belong. And if the numbers of seeds for all taxa are the same, e.g. 2 seeds are in the same node, one belongs to taxon A but the other one belongs to taxon B, all seeds are discarded.

Another semi-supervised version of GSOM was also developed by two of the co-authors, focusing on the creation of a GSOM classifier from data with up to 40% of missing labels and 25% of missing attribute values [34]. Two other methods are also conceptually related to S-GSOM. Seeded Region Growing (SRG) is a concept known in Computer Vision in which an image was grown from the known seeds (pixels or regions). For example, in Adams and Bischof [35], segmentation of images was achieved by incrementally assigning pixels to the seeds. Although the fundamental concept in SRG is similar to S-GSOM, there are the following major differences:

• The locations of the seeds in nodes are determined by the base clustering algorithm in S-GSOM, in contrast to SRG where the pixels are selected on the image as seeds. Therefore, seeds with the same label will all be connected in SRG whereas in S-GSOM they, and the resulting clusters, may scatter.

• S-GSOM compares the Euclidean distance between the border nodes of the clustered sets with its adjacent nodes of the un-clustered sets. SRG compares the mean grey value of the clustered sets with the surrounding pixels in the un-clustered sets.

• S-GSOM provides CP as the stop condition but SRG stops when all pixels have been allocated to clustered sets (equivalent to CP = 100% only).

The other related method is a semi-supervised SOM algorithm based on label propagation in a trained high resolution SOM [36]. It assigns a label vector, which contains the probabilistic memberships of the node in the available clusters, to each node. Batch training is performed by updating label vectors of all nodes through a probabilistic transition matrix, derived from distances between nodes. The algorithm stops when there is no change to any labels of unclassified nodes and all unlabelled nodes are classified to one of the clusters. This algorithm is similar to S-GSOM in that it also determines the unlabelled samples by propagating the label information from small amounts of labelled samples. However, the objectives of the two algorithms are different, leading to the following additional differences between the algorithms:

• The intention of S-GSOM is to stop the assignment before reaching the cluster boundaries, which is potentially the mixing region of classes in the application of binning. Alternatively, S-GSOM can be considered as a filtering algorithm to filter out the noisy sequences which were clustered by the normal GSOM. The label propagation of SOM stops when all nodes are clustered and the algorithm converges.

• The partial assignment feature of S-GSOM assists users to identify clusters without seeds (this will be discussed in the Results section), whereas the label propagation of SOM will form the clusters only with the given labelled samples, and thus cannot be used to identify sequences of unknown organisms.

• S-GSOM requires less computational power because it only operates on the surrounding nodes of clustered nodes in each step, whereas the label propagation of SOM updates all nodes in each iteration.

## Results

### Semi-supervised learning methods for binning

In order to provide an independent evaluation from the datasets used in Mavromatis et al. [7], we generated datasets that have different sizes, species and equal abundance to mimic multi-class datasets for testing other semi-supervised clustering algorithms on species separation. Genomic sequences were randomly sampled from the 488 completed microbial genomes available in the NCBI genome database (as of May 2007), which include both Archaea/Bacteria genomes [23]. The sampled prokaryotic sequences were cut into random fragments between 8 kb and 13 kb in length, and the tetranucleotide frequency of each fragment was computed (as outlined in the Methods section). A total of 7 datasets were created, three of which contain 10 species, another three contain 20 species and one contains 40 species [see Additional file 1].

**Figure 4:**
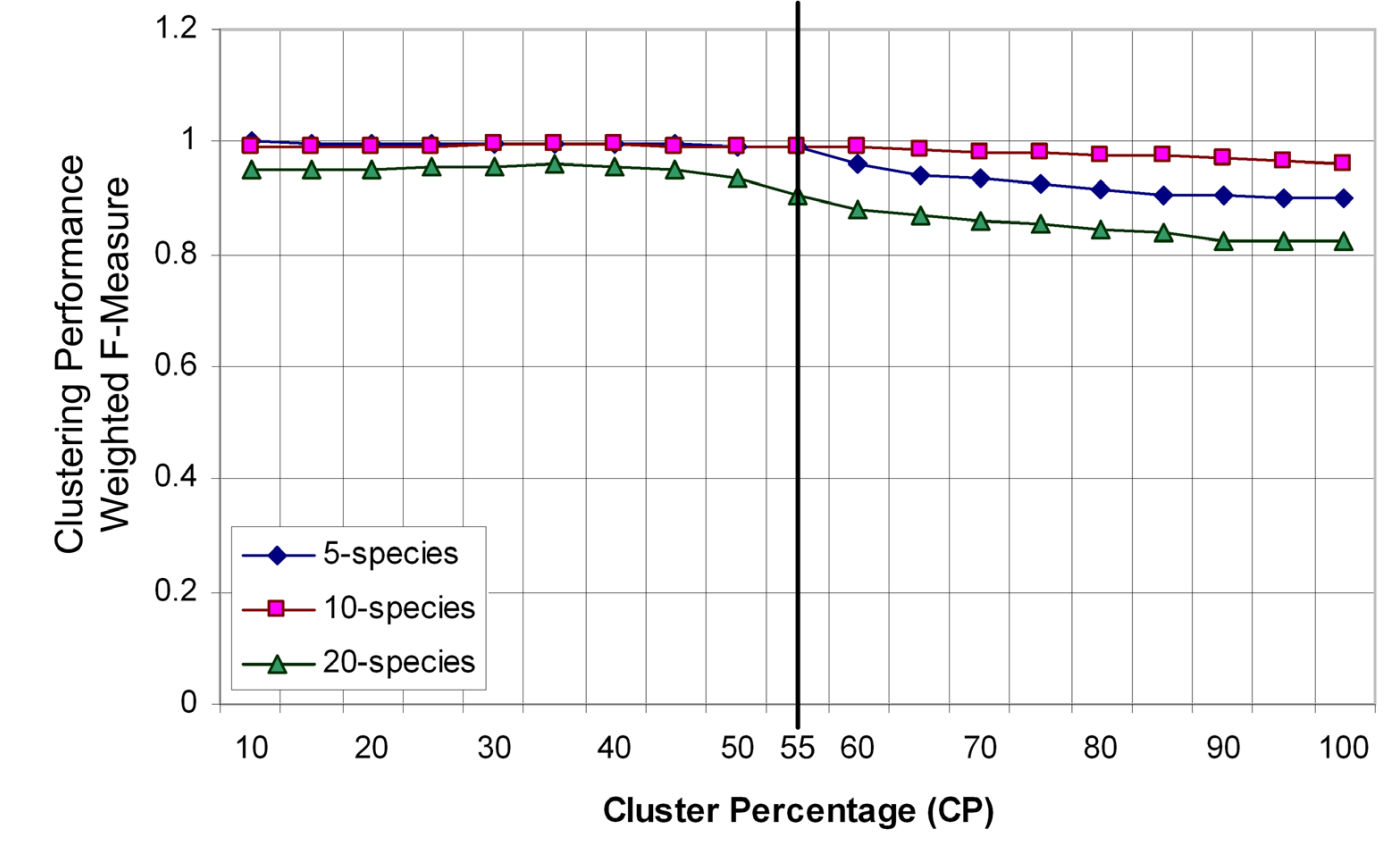
**Identification of an appropriate Clustering Percentage (CP)**. Five datasets for each of 5, 10 and 20 species are randomly sampled. The averages of S-GSOM's clustering performance for the datasets are plotted against Clustering Percentage (CP) values. A trend of decreasing in clustering performance with increasing CP can be noted. A compromised value of CP = 55% is marked where both the number of assigned nodes and clustering performance are high.

Four other well-known semi-supervised clustering algorithms, COP K-means [37], constrained K-means [38], seeded K-means [38] and the Transductive Support Vector Machine (TSVM) [39,40] [see Additional file 1], were used here alongside S-GSOM as a feasibility study of semi-supervised methods for binning metagenomic sequences. Some features of the proposed S-GSOM are explained here. Other semi-supervised algorithms are described in the literature. The choice of the user-defined parameter called Clustering Percentage (CP), used to specify the percentage of nodes that will be assigned to the seeded clusters relative to the total number of nodes in the feature map, was determined through experimental studies. As in our previous investigation [22] and in Abe et al. [13], it was identified that ambiguity of sequence fragments for closely related species occurred mostly at the cluster borders. An appropriate level of CP is necessary in order to avoid assigning too many sequence fragments and consequently incorrectly assigning fragments that are highly ambiguous. Preventing the algorithm from assigning ambiguous fragments is desirable, since it is very unlikely, even in a highly sophisticated procedure, to gather enough information to confidently distinguish the species of these highly ambiguous fragments. It was noted in our binning experiments that the clustering performance of S-GSOM declined when the CP was higher than 55% (Figure 4). While more than 80% of sequence fragments were assigned in most cases at CP = 55%, there was little reason for increasing the CP further to assign the remaining small amounts of fragments and taking the high risk of fragments being assigned inaccurately. Therefore, CP = 55% was used throughout the experiments in this paper. It should be noted that it is the intrinsic nature of GSOM that not all nodes have the same number of clustered sequence fragments. Furthermore, in the node assignment process of S-GSOM, nodes that clustered the highest density of sequence fragments tend to be assigned first. Therefore the amount of clustered sequences is not linearly proportional to the clustering percentage CP. A graph in Section 8 of the supplementary materials is provided to illustrate this [see Additional file 1]. One can also consider CP as a confidence threshold, analogous to the p-value in PhyloPythia, and opt to use a higher CP value to assign more contigs to bins with lower confidence, where a high CP value is equivalent to a low p-value in PhyloPythia.

The clustering performance of the semi-supervised algorithms were measured by two popular methods of evaluating multi-class clustering quality to ensure the validity of the results: Adjusted Rand Index [41] and Weighted F-measure [42] [see Additional file 1]. These methods compare the clustering performance of algorithms by the calculated indices. A higher index indicates a better clustering accuracy, and a larger difference between indexes means more significant results. The implementation and settings for semi-supervised algorithms were as below (source codes and programs for all in-house implemented algorithms are available by contacting the corresponding author):

1. COP K-means, Constrained K-means and Seeded K-means, which were implemented as described in literature, were trained with k-centres where k equals to the number of species in the dataset.

2. TSVM used the processing methods laid out for multi-class problems [40] and SVM-Light package [39,43].

3. S-GSOM was implemented as described in this paper. All results in this paper were obtained using the GSOM settings as specified in Section 7 of the supplementary materials [see Additional file 1].

In the above methods, different runs of random initialisation of the COP K-means and S-GSOM can lead to varied results. As Constrained K-means and Seeded K-means use the labelled sample for initialisation, there is no such issue with these methods. The results for COP K-means are the best results in 100 runs, with different random initialisation of the k cluster centres. Different initialisations for GSOM can result in different maps. However, the initialisation effect on GSOM is much less than on SOM, as GSOM starts with a minimum node structure in the beginning of training to allow for fast unfolding of the map; twisted maps are less common in GSOM [21]. Furthermore, different initialisation in GSOM will only have a minor effect on the final cluster formation for a well-ordered map. In all experiments reported in this paper, initialisation for all normalised dimensions was fixed at the mid value 0.5 to ensure repeatability.

**Figure 5:**
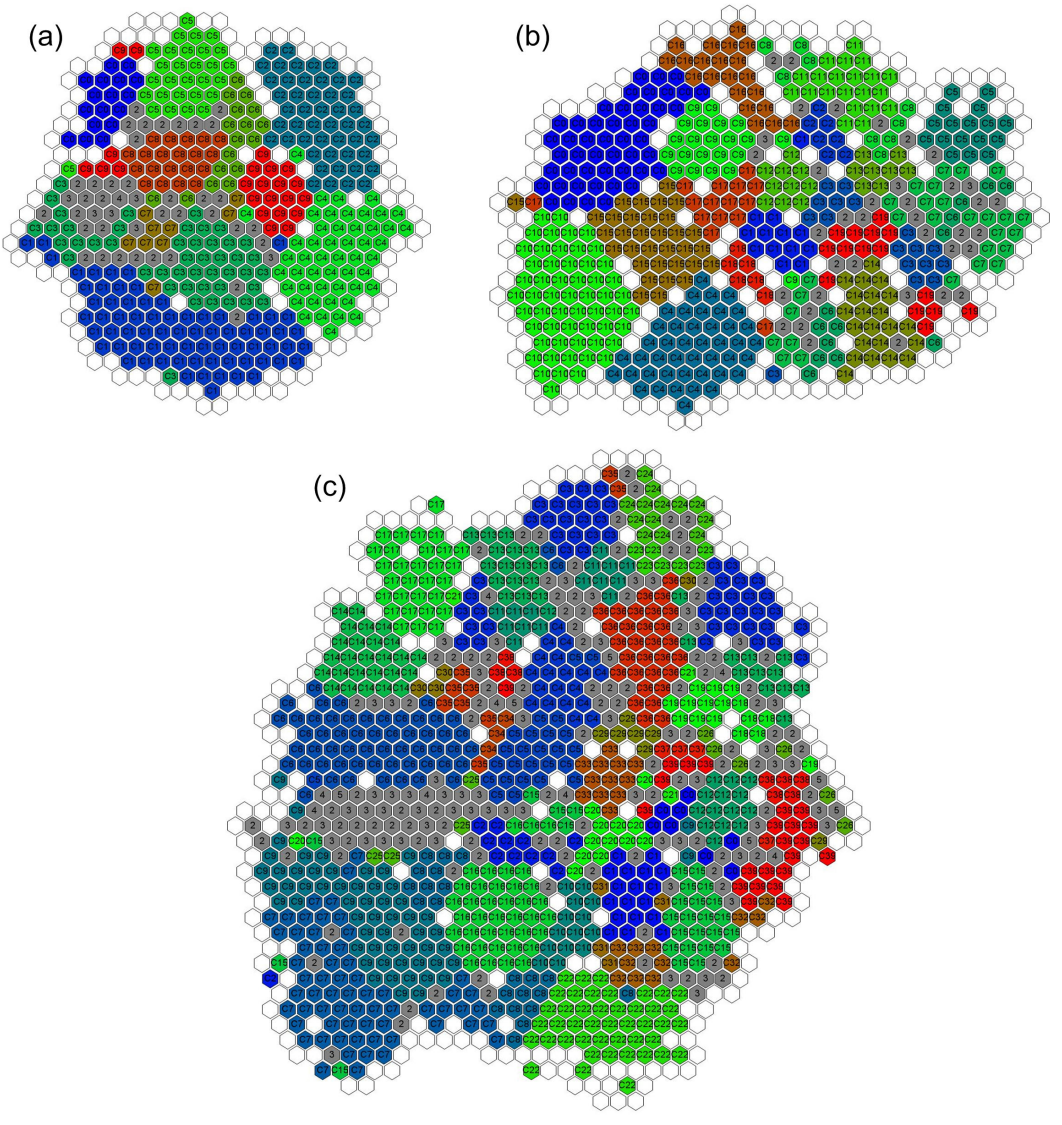
**Resulted GSOM maps of randomly sampled species**. The figure illustrates the GSOM results of clustering sequence fragments according to species: (a) 10Sp_Set1, (b) 20Sp_Set1 and (c) 40Sp_Set1. Each hexagon represents a single node. If it only contains a single species, it is displayed in a colour that uniquely identifies the species. A node without a letter means that there is no sample located in it. The grey node represents two or more species in the node and the number of species is displayed on the node.

The results of binning artificially generated fragments using different semi-supervised learning methods are tabulated in Table 1; the algorithm with the highest values of the two measures is shown in bold. The S-GSOM visualisation of binning sequences of 10Sp_Set1, 20Sp_Set1 and 40Sp_Set1 are provided in Figure 5.

**Table 1:** Clustering performance of semi-supervised algorithms. Performance is measured by the Adjusted Rand Index (ARI) and Weighted F-measure (WF). Results for COP K-means are the best results in 100 runs with different initial k cluster centres.

	COP K		Constrained K		Seeded K		TSVM		S-GSOM-55	
	
	ARI	WF	ARI	WF	ARI	WF	ARI	WF	ARI	WF
10Sp Set 1	0.84	0.94	0.84	0.94	0.84	0.93	0.25	0.59	**0.85**	**0.95**
10Sp Set 2	0.89	0.96	0.79	0.90	0.78	0.90	0.41	0.69	**0.93**	**0.97**
10Sp Set 3	0.58	0.83	**0.85**	**0.93**	0.84	**0.93**	0.27	0.62	0.83	**0.93**
20Sp Set 1	0.91	0.90	0.77	0.82	0.76	0.82	0.45	0.65	**0.97**	**0.96**
20Sp Set 2	0.76	0.82	0.70	0.79	0.67	0.79	0.43	0.62	**0.83**	**0.89**
20Sp Set 3	0.81	0.89	0.75	0.86	0.75	0.86	0.46	0.67	**0.97**	**0.98**
40Sp	0.58	0.76	0.71	0.85	0.68	0.84	0.24	0.56	**0.83**	**0.91**

S-GSOM showed consistently superior performance on both measures of clustering quality in all datasets tested, with the exception of Constrained K-means on the ARI measure for the 10Sp_Set3 dataset. TSVM has shown considerably worse performance among the algorithms. Although proposed as a semi-supervised method, TSVM is derived from the fully supervised algorithm SVM. Therefore, we suspect that insufficient labelled data significantly reduces its capability to classify correctly. The superior performance of S-GSOM is also attributed to the use of the variable CP, offering the flexibility of not assigning highly ambiguous fragments that are likely to overlap with other species. At CP = 55% between 75% and 90% of sequence fragments were assigned, whereas when all fragments were assigned (CP = 100%) the performance was similar to other algorithms. This shows S-GSOM's ability to filter out the noisy fragments to achieve better clustering performance.

We have also considered the 20-species datasets as an example to analyse the resolution of binning with S-GSOM. In the 20-species results, an average of 82% of fragments were assigned at CP = 55% and of these an average of 92% were correctly assigned to their seeds (species). The distribution of sequence fragments according to species is shown in Figure 5b. Nodes that contain fragments from more than one species are coloured grey and numbered with the number of species it represents. The fact that fragments that belong to different species were being clustered to the same node is attributed to the high similarity of nucleotide frequencies between very closely related species. For example, species NC_007146 (*Haemophilus influenzae *86-028NP) and NC_008309 (*Haemophilus somnus *129PT) are represented by nodes with labels 'C6' and 'C7' respectively. There was a significantly higher number of grey nodes at the boundary of these two species than of others. Further taxonomical examination of these two species revealed that they are phylogenetically close and are in the same family, *Pasteurellaceae*. It is also identified that all seeds of these species are located near the boundaries of clusters. This further highlights the importance of obtaining seeds in non-boundary regions.

**Figure 6:**
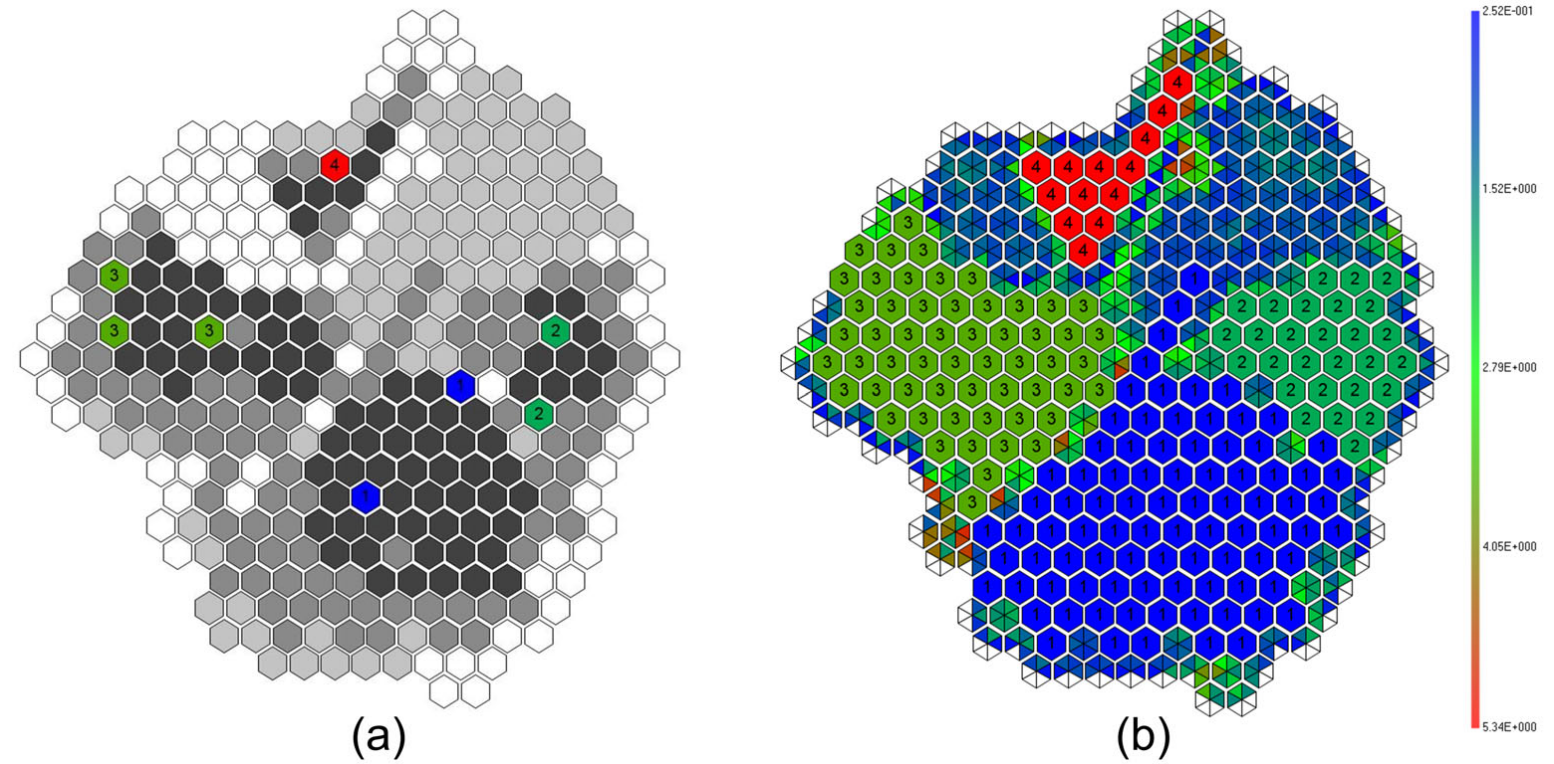
**Illustration of exploring an unseeded cluster**. (a) The 5-species S-GSOM map. The seeded nodes are shown with unique colours and labels. Nodes in charcoal colour represent nodes that will be assigned when CP = 27% and dark grey nodes at CP = 55%, light grey at CP = 77%, and white at CP = 100%. (b) Inter-node distance map with nodes assigned at CP = 55%.

A prominent advantage of the proposed seeding method compared with other semi-supervised clustering algorithms is the ability to identify a small number of species that do not have any fragment that qualifies as a seed. In order to demonstrate this advantage, an iso-CP (constant CP contours) plot is shown in Figure 6a, generated with sequence fragments from 5 species (for clarity of presentation) in which there is one unseeded species and the seeds of other species are represented by unique colours. Nodes in charcoal colour represent nodes that will be assigned when CP = 27% and dark grey nodes at the recommended value of CP = 55%, light grey at CP = 77%, and white at CP = 100%, respectively. Figure 6b shows the allocation of nodes to seeds at CP = 55%, where it can be seen together with Figure 6a that there is a largely unassigned region at this point, and increasing the CP to 77% will result in a rapid assignment of many nodes. This situation is most likely when a species is relatively abundant, but does not have a seed to allocate nodes to. However, incorrect assignment of nodes can sometimes occur at a low CP. For instance, Figure 6b shows a protrusion of species '1' into the unassigned region, which actually belongs to species '5' that does not have any seed.

### Binning of sequence fragments in simulated metagenomic datasets

Three simulated metagenomic datasets, which vary in relative abundance and number of species, were created by Mavromatis et al. [7] and placed in the FAMeS database [44] to facilitate benchmarking of metagenomic data processing methods, which include, but are not limited to, binning methods. The sequence fragments in the simulated datasets were assembled using three commonly used sequence assembling programs, Arachne [45], Phrap [46] and JAZZ [47] at the U.S. Department of Energy, Joint Genome Institute. In this section of the experiment, we test the performance of S-GSOM in binning against three binning methods: BLAST, *k*-mer and PhyloPythia, reported on the datasets [see Additional file 1].

The three simulated datasets can be designated as being Low Complexity, Medium Complexity or High Complexity. The Low Complexity (simLC) dataset is dominated by one near-clonal species together with a few low-abundance ones, which simulates a microbial community in an environment such as a bioreactor [48,49]. The Medium Complexity (simMC) dataset mimics a community structure similar to an acid mine drainage biofilm [1], which consists of more than one dominant species flanked by low-abundance ones. The High Complexity (simHC) dataset, on the other hand, does not include any dominant populations.

Two considerations were taken into account when comparing the reported binning results with S-GSOM. Firstly, the results in [7] showed that sequence reads assembled by JAZZ produce a very small number of binned contigs compared to the other two assemblers Arachne and Phrap, which greatly diminishes the purpose of binning. Therefore, fragments assembled by JAZZ are excluded from our analysis. Secondly, the simHC dataset resembles a complex community structure that can be common in reality and a well-performing binning method is therefore highly desirable. However, a complex community that has no dominant populations has insufficient DNA sequences of the same species to form longer contigs. As reported in literature [7,16], composition-based analysis requires a sufficiently long sequence length to ensure the accuracy of binning. Therefore, the simHC dataset is also excluded from our analysis.

Like other binning methods, S-GSOM can form bins at different taxonomic levels. Bins that have different labels in a lower taxonomic level may belong to the same higher taxonomic level and can be combined to form higher taxonomic bins, thus accuracy is higher but at the cost of lower taxonomic resolution. For the purpose of fair comparison, all methods need to be compared at the same taxonomic level of binning. Binning at a very high level clearly has no significance, therefore the results are compared at the order level here and results for comparing at other taxonomic levels are included in the supplementary materials [see Additional file 1].

Two confidence settings of S-GSOM (high confidence – CP = 55% and low confidence – CP = 75%) were tested on the simulated metagenomic data, which correspond to two different settings of PhyloPythia. The S-GSOMs were trained using parameters specified in Section 7 of the supplementary materials and the final feature maps are also provided there. The binning results of S-GSOM, PhyloPythia, *k-*mer and BLAST on the simLC (Tables 2 and 4) and the simMC (Tables 3 and 5) datasets are reported here. At the order level, the results for a different complexity dataset are shown in two separate tables, one for binning contigs greater and equal to 8 kb length and one for binning contigs with at least 10 reads. Details of the datasets at different taxonomic levels are also provided in Section 1 of the supplementary materials. Performance evaluation results at class level and family level are included in Section 6 of the supplementary materials and all contig assignments can be downloaded from S-GSOM homepage [50]. Note that the published results in Mavromatis et al. used simple averages of all bins regardless of their sizes (number of contigs in a bin). Here we use a weighted average that gives higher weighting to larger bins to better reflect the amount of correctly binned contigs. The exact step of performance evaluation is provided in Section 5 of the supplementary materials.

**Table 2:** Binning summary for Low Complexity datasets for contigs larger than 8 kb.

Assembler	Method	Bins	Binned Contigs	Total #Contigs	%of BinContigs	#ofPred NotInAct	wSp	wSn
Arachne	kmer (7 mer)	0	0	202	0	85	-	0.000
Arachne	kmer (8 mer)	0	0	202	0	149	-	0.000
Arachne	BLAST distr 1	0	0	202	0	0	-	0.000
Arachne	BLAST distr 2	0	0	202	0	0	-	0.000
Arachne	S-GSOM (CP = 55%)	1	141	202	69.8	0	1.000	0.698
Arachne	gen PhyloPythia (p:0.85)	1	168	202	83.17	0	1.000	0.832
Arachne	ssp PhyloPythia (p:0.85)	1	186	202	92.08	0	1.000	0.921
Arachne	S-GSOM (CP = 75%)	1	180	202	89.11	0	1.000	0.891
**Arachne**	**gen PhyloPythia (p:0.5)**	**1**	**201**	**202**	**99.5**	**0**	**1.000**	**0.995**
**Arachne**	**ssp PhyloPythia (p:0.5)**	**1**	**201**	**202**	**99.5**	**0**	**1.000**	**0.995**
Phrap	kmer (7 mer)	0	0	229	0	129	-	0.000
Phrap	kmer (8 mer)	0	0	229	0	154	-	0.000
Phrap	BLAST distr 1	0	0	229	0	0	-	0.000
Phrap	BLAST distr 2	0	0	229	0	0	-	0.000
Phrap	S-GSOM (CP = 55%)	1	157	229	68.56	0	1.000	0.686
Phrap	gen PhyloPythia (p:0.85)	1	185	229	80.79	0	1.000	0.808
Phrap	ssp PhyloPythia (p:0.85)	1	205	229	89.52	0	1.000	0.895
Phrap	S-GSOM (CP = 75%)	1	204	229	89.08	0	1.000	0.891
**Phrap**	**gen PhyloPythia (p:0.5)**	**1**	**227**	**229**	**99.13**	**0**	**1.000**	**0.991**
**Phrap**	**ssp PhyloPythia (p:0.5)**	**1**	**227**	**229**	**99.13**	**0**	**1.000**	**0.991**

Total#Contigs: Total number of contigs in the dataset; %ofBinContigs: The percentage of contigs binned; #ofPredNotInAct: The number of contigs predicted as a taxon that is not present in the dataset, which are treated as the un-binned contigs; wSp: Weighted specificity; wSn: Weighted sensitivity.

**Table 3:** Binning summary for Medium Complexity datasets for contigs larger than 8 kb.

Assembler	Method	Bins	Binned Contigs	Total #Contigs	%of BinContigs	#ofPred NotInAct	wSp	wSn
Arachne	kmer (7 mer)	0	0	301	0	47	-	0.000
Arachne	kmer (8 mer)	0	0	301	0	191	-	0.000
Arachne	BLAST distr 1	0	0	301	0	0	-	0.000
Arachne	BLAST distr 2	0	0	301	0	0	-	0.000
Arachne	S-GSOM (CP = 55%)	2	220	301	73.09	0	1.000	0.731
Arachne	gen PhyloPythia (p:0.85)	2	242	301	80.4	0	1.000	0.804
Arachne	ssp PhyloPythia (p:0.85)	2	242	301	80.4	0	1.000	0.804
Arachne	S-GSOM (CP = 75%)	2	279	301	92.69	0	1.000	0.927
**Arachne**	**gen PhyloPythia (p:0.5)**	**2**	**301**	**301**	**100**	**0**	**1.000**	**1.000**
**Arachne**	**ssp PhyloPythia (p:0.5)**	**2**	**301**	**301**	**100**	**0**	**1.000**	**1.000**
Phrap	kmer (7 mer)	0	0	401	0	84	-	0.000
Phrap	kmer (8 mer)	0	0	401	0	271	-	0.000
Phrap	BLAST distr 1	0	0	401	0	0	-	0.000
Phrap	BLAST distr 2	0	0	401	0	0	-	0.000
Phrap	S-GSOM (CP = 55%)	2	318	401	79.3	0	1.000	0.793
Phrap	gen PhyloPythia (p:0.85)	2	301	401	75.06	0	1.000	0.751
Phrap	ssp PhyloPythia (p:0.85)	2	295	401	73.57	0	1.000	0.736
Phrap	S-GSOM (CP = 75%)	2	367	401	91.52	0	1.000	0.915
**Phrap**	**gen PhyloPythia (p:0.5)**	**2**	**399**	**401**	**99.5**	**1**	**1.000**	**0.995**
**Phrap**	**ssp PhyloPythia (p:0.5)**	**2**	**399**	**401**	**99.5**	**1**	**1.000**	**0.995**

**Table 4:** Binning summary for Low Complexity datasets for contigs with at least 10 reads.

Assembler	Method	Bins	Binned Contigs	Total #Contigs	%of BinContigs	#ofPred NotInAct	wSp	wSn
Arachne	kmer (7 mer)	0	0	367	0	168	-	0.000
Arachne	kmer (8 mer)	0	0	367	0	312	-	0.000
Arachne	BLAST distr 1	0	0	367	0	0	-	0.000
Arachne	BLAST distr 2	0	0	367	0	0	-	0.000
Arachne	S-GSOM (CP = 55%)	3	295	367	80.38	0	1.000	0.798
Arachne	gen PhyloPythia (p:0.85)	2	214	367	58.31	0	1.000	0.583
Arachne	ssp PhyloPythia (p:0.85)	2	236	367	64.31	0	1.000	0.638
**Arachne**	**S-GSOM (CP = 75%)**	**3**	**343**	**367**	**93.46**	**0**	**0.950**	**0.926**
Arachne	gen PhyloPythia (p:0.5)	2	292	367	79.56	0	1.000	0.796
Arachne	ssp PhyloPythia (p:0.5)	2	296	367	80.65	0	1.000	0.798
Phrap	kmer (7 mer)	2	3	482	0.62	159	1.000	0.000
Phrap	kmer (8 mer)	3	17	482	3.53	281	1.000	0.000
Phrap	BLAST distr 1	0	0	482	0	0	-	0.000
Phrap	BLAST distr 2	0	0	482	0	1	-	0.000
Phrap	S-GSOM (CP = 55%)	8	381	482	79.05	9	1.000	0.728
Phrap	gen PhyloPythia (p:0.85)	3	236	482	48.96	0	1.000	0.488
Phrap	ssp PhyloPythia (p:0.85)	3	272	482	56.43	0	1.000	0.560
**Phrap**	**S-GSOM (CP = 75%)**	**8**	**443**	**482**	**91.91**	**9**	**1.000**	**0.840**
Phrap	gen PhyloPythia (p:0.5)	4	368	482	76.35	1	1.000	0.759
Phrap	ssp PhyloPythia (p:0.5)	5	387	482	80.29	1	1.000	0.797

**Table 5:** Binning summary for Medium Complexity datasets for contigs with at least 10 reads.

Assembler	Method	Bins	Binned Contigs	Total #Contigs	%of BinContigs	#ofPred NotInAct	wSp	wSn
Arachne	kmer (7 mer)	1	2	1372	0.15	133	1.000	0.000
Arachne	kmer (8 mer)	0	0	1372	0	1241	-	0.000
Arachne	BLAST distr 1	0	0	1372	0	0	-	0.000
Arachne	BLAST distr 2	0	0	1372	0	1	-	0.000
Arachne	S-GSOM (CP = 55%)	5	1061	1372	77.33	0	0.998	0.768
Arachne	gen PhyloPythia (p:0.85)	3	562	1372	40.96	0	1.000	0.409
Arachne	ssp PhyloPythia (p:0.85)	3	657	1372	47.89	0	1.000	0.478
**Arachne**	**S-GSOM (CP = 75%)**	**5**	**1253**	**1372**	**91.33**	**0**	**0.983**	**0.897**
Arachne	gen PhyloPythia (p:0.5)	4	1036	1372	75.51	6	1.000	0.753
Arachne	ssp PhyloPythia (p:0.5)	4	1102	1372	80.32	4	1.000	0.802
Phrap	kmer (7 mer)	1	1	1980	0.05	163	1.000	0.000
Phrap	kmer (8 mer)	2	391	1980	19.75	1457	1.000	0.000
Phrap	BLAST distr 1	0	0	1980	0	2	-	0.000
Phrap	BLAST distr 2	0	0	1980	0	3	-	0.000
Phrap	S-GSOM (CP = 55%)	8	1409	1980	71.16	9	0.995	0.686
Phrap	gen PhyloPythia (p:0.85)	3	799	1980	40.35	1	1.000	0.404
Phrap	ssp PhyloPythia (p:0.85)	3	844	1980	42.63	1	1.000	0.426
**Phrap**	**S-GSOM (CP = 75%)**	**8**	**1708**	**1980**	**86.26**	**9**	**0.991**	**0.816**
Phrap	gen PhyloPythia (p:0.5)	5	1484	1980	74.95	6	1.000	0.745
Phrap	ssp PhyloPythia (p:0.5)	5	1524	1980	76.97	4	1.000	0.767

The results in Table 2 and 3 showed that S-GSOM performed reasonably for binning contigs longer than 8 kb, where it is more accurate than all settings of *k-*mer and BLAST binning methods, but was outperformed by PhyloPythia in both confidence settings (CP = 75% vs p = 0.5 and CP = 55% vs p = 0.85) regardless of data complexity and the assembler used. Nevertheless, S-GSOM still performed better than PhyloPythia for the simMC, particularly in terms of sensitivity, i.e. having a higher true positive rate, when the reference taxonomic level was at the family level (see Section 6(ii) of the supplementary materials). While PhyloPythia performed best for all ≥ 8 kb tests, the S-GSOM binning method was the best-performing method when used to bin contigs that have at least 10 reads (Tables 4 and 5).

Considering the fact that S-GSOM is the only one amongst the tested binning methods that does not use any knowledge of completed genomes, this performance has demonstrated its feasibility, particularly for analysing metagenomic communities that contain a number of unknown organisms that lack similarity to known genomes. S-GSOM has proved itself competitive with the most sophisticated binning algorithm, PhyloPythia, over which S-GSOM has an advantage in being easier and faster to build because it does not require the use of reference genomes for training.

## Discussion and conclusion

Recently, the importance of environmental genomics has been recognised [51,52] and more environmental sequencing projects are being undertaken [1-3,18]. Most of the current methods of binning compare a sequence fragment to a reference set of genomes. Such methods tend to have high assignment accuracy particularly when a strong similarity exists between the reference and environmental genomes, either at the compositional or sequence level. However, the performance declines drastically if the best match found is still quite different from the one being queried. On the contrary, extracting reference sequences from within the metagenome, as in S-GSOM, will ensure better matching. It also facilitates a means of exploring species that have not yet been identified. In fact, such reference information from longer assembled contigs was used in the binning process of two prominent metagenomic projects for low-diversity microbial communities [1,4].

S-GSOM enables the clustering of sequence fragments, with phylogenetical meaning, by using very small amounts of sequence fragments around the highly conserved genes as seeds. It has several advantages over other unsupervised and semi-supervised clustering algorithms. First and most importantly, due to its visualisation property, S-GSOM allows the user to visually identify one or two relatively abundant species that do not have any seed, as demonstrated earlier, by using CP contour display together with inter-node distances. However, to identify unseeded clusters, it is necessary to have a larger number of fragments in the unseeded cluster, at least as many as the seeded clusters, i.e. the species is relatively abundant. If the unseeded clusters have far less samples than the seeded clusters, the unseeded cluster may be assigned to the other clusters at low CP values, or be considered as part of the border of neighbouring clusters and thus become hardly detectable. On the other hand, if the unseeded clusters have far more samples than the seeded clusters, the clustering percentage needs to be set to a lower value, as otherwise there will be more incorrectly assigned samples.

The other advantages of S-GSOM include: 1) sequences can easily be reassigned without retraining by varying the CP that is directly related to confident assignation, 2) the ability to function as a fully automated clustering process, 3) the potential use as a visualisation aid for identifying unseeded clusters with iso-CP contours and inter-node distances, and 4) this technique can easily be applied to other applications when the labelled data is very limited but a lot of unlabelled data is available.

For the discovery of sequences of unknown microorganisms using S-GSOM, it should be expected that seeds may or may not be available. When seeds are available, S-GSOM allows the identification of relationships between the seed-associated clusters based on phylogenetic analysis of 16S rRNA sequences. If the phylogenetic analysis detects any unknown microorganism, we will be able to discover the sequences associated with this organism from its 16S rRNA sequences using S-GSOM. This function will be of great assistance in sorting sequences in metagenomic datasets according to phylogenetic relationships. However, if no seed is available, it means that there is no 16S rRNA sequence in the datasets for assessing the phylogenetic relationships of this cluster. In such circumstances, we can still obtain the sequences in the possible bins, which were identified by using the iso-CP contour map, then compare the sequences with existing databases by BLAST searching. If any marker gene is detected, such as elongation factors and/or cytochrome oxidase, then we may assess whether these sequences are from unknown or known microorganisms by phylogenetic analysis.

In the results section, we noted a major requirement for the optimal performance of S-GSOM. As the proposed algorithm uses seeds to assign more unknown fragments, poor seeds that are at the boundary of clusters, which are ambiguous in themselves, can greatly affect the resulting assignment. This problem can possibly be solved by observing the data distribution density around the seeds. For instance, if a seed occupies a single node and its surrounding nodes also contain fewer samples, the seed has a high chance of being a poor seed. While a choice of CP = 55% yielded accurate sequence assignment in the two separate tests, a user can opt to use a lower clustering percentage (e.g. CP = 35%) for more confident assignment at the cost of fewer sequences being assigned. Conversely, using a higher CP value (e.g. CP = 75%) will assign more fragments but risk incorrect assignments. This feature is also evident in the tests that use simulated metagenomic data, where S-GSOM traded specificity for an increase in sensitivity.

Although these composition-based binning methods have shown good results, currently they are hindered by the requirement of long sequence length. This limitation of length is partially due to the occurrence of chimeric sequences from cloning procedures of experiments and from the incorrect assembly of sequences. The former source of chimeric sequences can be reduced by improving the sequence strategy, such as using a Roche 454 genome sequencer FLX, which excludes sequence cloning and hence generates less chimeric sequences. The latter source of chimeric sequences is caused by the incompatible design of the currently available assemblers. These assemblers are commonly designed to assemble all reads into one single genome. However, this application does not satisfy the requirement of metagenomic datasets which are of poor coverage depth and contain multiple genomes. As a result, it leads to the occurrence of chimeric sequences when highly conserved stretches of sequences, e.g. transposases, are shared by multiple species or strains. Mavromatis et al. [7] tested the simulated metagenomic datasets and showed that the numbers of chimeric sequence became significant for the assembled short sequences, e.g. < 8 kb, and led to the low quality of binning. Therefore, if the number of chimeric sequences is reduced, the required sequence length can also be reduced. To help the reduction of chimeric sequences, we suggest including the compositional information in the assembling level.

## Authors' contributions

CKKC designed the overall study, implemented the algorithm and performed analysis. ALH contributed to the design of the experiments and interpretation and analysis of results. SLT designed the algorithm with CKKC and biological aspects of the study. All authors contributed to the preparation of the manuscript, also read and approved the final manuscript.

## Supplementary Material

Additional File 1 **Supplementary materials**. This file is in pdf format. Section 1 provides details of all datasets used in this article. Section 2 gives descriptions of the semi-supervised algorithms, COP K-Means, Constrained K-Means, Seeded K-Means and Transductive SVM, used in the comparison with S-GSOM. Section 3 describes two popular clustering quality measures, the Adjusted Rand Index and Weighted F-Measure, in detail. Sections 4–7 provide more information on the experiments with simulated metagenomic datasets. Section 4 briefly describes the three binning algorithms, BLAST, k-mer and PhyloPythia. Section 5 describes the steps used to evaluate binning results of methods. Section 6 shows the detailed results of S-GSOM for all individual bins at different taxonomic levels for the simulated metagenomic dataset tests. Section 7 provides the training parameters used for S-GSOM and the resultant trained GSOM maps. Section 8 shows an example graph to demonstrate the relationship between CP value and the percentage of assigned samples. Finally, Section 9 lists the references used in the supplementary material. Click here for file
