# Supplementary material for: Binning sequences using very sparse labels within a metagenome
Source: BMC Bioinformatics. 2008 Apr 28;9:215. doi: 10.1186/1471-2105-9-215 (PMC2383919; doi:10.1186/1471-2105-9-215)
Supplement: Additional File 1 — Supplementary materials. This file is in pdf format. Section 1 provides details of all datasets used in this article. Section 2 gives descriptions of the semi-supervised algorithms, COP K-Means, Constrained K-Means, Seeded K-Means and Transductive SVM, used in the comparison with S-GSOM. Section 3 describes two popular clustering quality measures, the Adjusted Rand Index and Weighted F-Measure, in detail. Sections 4–7 provide more information on the experiments with simulated metagenomic datasets. Section 4 briefly describes the three binning algorithms, BLAST, k-mer and PhyloPythia. Section 5 describes the steps used to evaluate binning results of methods. Section 6 shows the detailed results of S-GSOM for all individual bins at different taxonomic levels for the simulated metagenomic dataset tests. Section 7 provides the training parameters used for S-GSOM and the resultant trained GSOM maps. Section 8 shows an example graph to demonstrate the relationship between CP value and the percentage of assigned samples. Finally, Section 9 lists the references used in the supplementary material. [file 1471-2105-9-215-S1.pdf]

Supplementary Materials

Table of Contents

1. Dataset details 2
   i. Lists of All Species in Generated Datasets 2
   ii. Details of the simulated benchmark datasets 7
      Details of datasets at family level 7
      Details of datasets at order level 8
      Details of datasets at class level 9
2. Semi-Supervised Clustering Algorithms 10
3. Clustering Performance Measures 12
4. Binning Algorithms 14
5. Result evaluation procedures 15
6. Result comparisons of different methods at different taxonomic level for the simulated benchmark datasets 18
   i. Family level, >=8 kb, simLC 19
   ii. Family level, >=8 kb, simMC 20
   iii. Family level, >=10 reads, simLC 21
   iv. Family level, >=10 reads, simMC 22
   v. Order level, >=8 kb, simLC 23
   vi. Order level, >=8 kb, simMC 24
   vii. Order level, >=10 reads, simLC 25
   viii. Order level, >=10 reads, simMC 26
   ix. Class level, >=8 kb, simLC 27
   x. Class level, >=8 kb, simMC 28
   xi. Class level, >=10 reads, simLC 29
   xii. Class level, >=10 reads, simMC 30
7. GSOM settings and GSOM maps 31
8. Relationship between CP and the percentage of assigned samples 35
9. References 36

1. Dataset details

i. Lists of All Species in Generated Datasets

Table 1: List of Species in 10Sp-Set1

NCBI Accession Number	Species Name	Genome Length
NC_002162	*Ureaplasma parvum* serovar 3 str. ATCC 700970	751716
NC_002516	*Pseudomonas aeruginosa* PAO1	6264400
NC_002937	*Desulfovibrio vulgaris* subsp. *vulgaris* str. Hildenborough	3570855
NC_002947	*Pseudomonas putida* KT2440	6181860
NC_003047	*Sinorhizobium meliloti* 1021	3654132
NC_003901	*Methanosarcina mazei* Go1	4096342
NC_004547	*Erwinia carotovora* subsp. *atroseptica* SCRI1043	5064016
NC_004578	*Pseudomonas syringae* pv. *tomato* str. DC3000	6397123
NC_006370	*Photobacterium profundum* SS9	4085301
NC_006510	*Geobacillus kaustophilus* HTA426	3544773

Table 2: List of Species in 10Sp-Set2

NCBI Accession Number	Species Name	Genome Length
NC_005085	*Chromobacterium violaceum* ATCC 12472	4751080
NC_002935	*Corynebacterium diphtheriae* NCTC 13129	2488635
NC_006512	*Idiomarina loihiensis* L2TR	2839318
NC_007626	*Magnetospirillum magneticum* AMB-1	4967148
NC_008254	*Mesorhizobium* sp. BNC1	4412446
NC_007955	*Methanococcoides burtonii* DSM 6242	2575032
NC_007947	*Methylobacillus flagellatus* KT	2971517
NC_003485	*Streptococcus pyogenes* MGAS8232	1895017
NC_007181	*Sulfolobus acidocaldarius* DSM 639	2225959
NC_002967	*Treponema denticola* ATCC 35405	2843201

Table 3: List of Species in 10Sp-Set3

NCBI Accession Number	Species Name	Genome Length
NC_007760	*Anaeromyxobacter dehalogenans* 2CP-C	5013479
NC_006347	*Bacteroides fragilis* YCH46	5277274
NC_002163	*Campylobacter jejuni* subsp. *jejuni* NCTC 11168	1641481
NC_006677	*Gluconobacter oxydans* 621H	2702173
NC_008242	*Mesorhizobium* sp. BNC1	343931
NC_008254	*Mesorhizobium* sp. BNC1	4412446
NC_004432	*Mycoplasma penetrans* HF-2	1358633
NC_007492	*Pseudomonas fluorescens* PfO-1	6438405
NC_005773	*Pseudomonas syringae* pv. *phaseolicola* 1448A	5928787
NC_004337	*Shigella flexneri* 2a str. 301	4607203
NC_003919	*Xanthomonas axonopodis* pv. *citri* str. 306	5175554

Table 4: List of Species in 20Sp-Set1

NCBI Accession Number	Species Name	Genome Length
NC_009348	*Aeromonas salmonicida* subsp. *salmonicida* A449	4702402
NC_000918	*Aquifex aeolicus* VF5	1551335
NC_007716	Aster yellows witches'-broom phytoplasma AYWB	706569
NC_008783	*Bartonella bacilliformis* KC583	1445021
NC_002696	*Caulobacter crescentus* CB15	4016947
NC_003361	*Chlamydophila caviae* GPIC	1173390
NC_007146	*Haemophilus influenzae* 86-028NP	1913428
NC_008309	*Haemophilus somnus* 129PT	2007700
NC_006055	*Mesoplasma florum* L1	793224
NC_007644	*Moorella thermoacetica* ATCC 39073	2628784
NC_002755	*Mycobacterium tuberculosis* CDC1551	4403837
NC_005364	*Mycoplasma mycoides* subsp. *mycoides* SC str. PG1	1211703
NC_002950	*Porphyromonas gingivalis* W83	2343476
NC_005072	*Prochlorococcus marinus* subsp. *pastoris* str. CCMP1986	1657990
NC_007969	*Psychrobacter cryohalolentis* K5	3059876
NC_003155	*Streptomyces avermitilis* MA-4680	9025608

NC_000911	*Synechocystis* sp. PCC 6803	3573470
NC_007759	*Syntrophus aciditrophicus* SB	3179300
NC_000853	*Thermotoga maritima* MSB8	1860725
NC_006840	*Vibrio fischeri* ES114	2906179

Table 5: List of Species in 20Sp-Set2

NCBI Accession Number	Species Name	Genome Length
NC_008782	*Acidovorax* sp. JS42	4448856
NC_006274	*Bacillus cereus* E33L	5300915
NC_001318	*Borrelia burgdorferi* B31	910724
NC_008599	*Campylobacter fetus* subsp. *fetus* 82-40	1773615
NC_008278	*Frankia alni* ACN14a	7497934
NC_002662	*Lactococcus lactis* subsp. *lactis* Il1403	2365589
NC_008011	*Lawsonia intracellularis* PHE/MN1-00	1457619
NC_005823	*Leptospira interrogans* serovar Copenhageni str. Fiocruz L1-130	4277185
NC_008531	*Leuconostoc mesenteroides* subsp. *mesenteroides* ATCC 8293	2038396
NC_006300	*Mannheimia succiniciproducens* MBEL55E	2314078
NC_007681	*Methanosphaera stadtmanae* DSM 3091	1767403
NC_007644	*Moorella thermoacetica* ATCC 39073	2628784
NC_004432	*Mycoplasma penetrans* HF-2	1358633
NC_007925	*Rhodopseudomonas palustris* BisB18	5513844
NC_007384	*Shigella sonnei* Ss046	4825265
NC_007622	*Staphylococcus aureus* RF122	2742531
NC_002976	*Staphylococcus epidermidis* RP62A	2616530
NC_007168	*Staphylococcus haemolyticus* JCSC1435	2685015
NC_003028	*Streptococcus pneumoniae* TIGR4	2160842
NC_006833	*Wolbachia endosymbiont* strain TRS of *Brugia malayi*	1080084

Table 6: List of Species in 20Sp-Set3

NCBI Accession Number	Species Name	Genome Length
NC_007618	*Brucella melitensis* biovar Abortus 2308	2121359
NC_002935	*Corynebacterium diphtheriae* NCTC 13129	2488635
NC_002971	*Coxiella burnetii* RSA 493	1995281
NC_008571	*Gramella forsetii* KT0803	3798465
NC_008212	*Haloquadratum walsbyi* DSM 16790	3132494
NC_009135	*Methanococcus maripaludis* C5	1780761
NC_003552	*Methanosarcina acetivorans* C2A	5751492
NC_008596	*Mycobacterium smegmatis* str. MC2 155	6988209
NC_000962	*Mycobacterium tuberculosis* H37Rv	4411532
NC_002771	*Mycoplasma pulmonis* UAB CTIP	963879
NC_007614	*Nitrosospira multiformis* ATCC 25196	3184243
NC_005126	*Photorhabdus luminescens* subsp. *laumondii* TTO1	5688987
NC_008027	*Pseudomonas entomophila* L48	5888780
NC_007954	*Shewanella denitrificans* OS217	4545906
NC_007613	*Shigella boydii* Sb227	4519823
NC_008022	*Streptococcus pyogenes* MGAS10270	1928252
NC_007516	*Synechococcus* sp. CC9605	2510659
NC_007575	*Thiomicrospira denitrificans* ATCC 33889	2201561
NC_005139	*Vibrio vulnificus* YJ016	3354505
NC_006833	*Wolbachia endosymbiont* strain TRS of *Brugia malayi*	1080084

Table 7: List of Species in 40Sp-Set1

NCBI Accession Number	Species Name	Genome Length
NC_008260	*Alcanivorax borkumensis* SK2	3120143
NC_008541	*Arthrobacter* sp. FB24	4698945
NC_006513	*Azoarcus* sp. EbN1	4296230
NC_003997	*Bacillus anthracis* str. Ames	5227293
NC_006582	*Bacillus clausii* KSM-K16	4303871
NC_006322	*Bacillus licheniformis* ATCC 14580	4222645
NC_004663	*Bacteroides thetaiotaomicron* VPI-5482	6260361
NC_002928	*Bordetella parapertussis* 12822	4773551

NC_002696	*Caulobacter crescentus* CB15	4016947
NC_005085	*Chromobacterium violaceum* ATCC 12472	4751080
NC_001263	*Deinococcus radiodurans* R1	2648638
NC_000907	*Haemophilus influenzae* Rd KW20	1830138
NC_008529	*Lactobacillus delbrueckii* subsp. *bulgaricus* ATCC BAA-365	1856951
NC_008555	*Listeria welshimeri* serovar 6b str. SLCC5334	2814130
NC_000909	*Methanocaldococcus jannaschii* DSM 2661	1664970
NC_002977	*Methylococcus capsulatus* str. Bath	3304561
NC_009077	*Mycobacterium* sp. JLS	6048425
NC_007633	*Mycoplasma capricolum* subsp. *capricolum* ATCC 27343	1010023
NC_000912	*Mycoplasma pneumoniae* M129	816394
NC_007481	*Pseudoalteromonas haloplanktis* TAC125	3214944
NC_002947	*Pseudomonas putida* KT2440	6181863
NC_008709	*Psychromonas ingrahamii* 37	4559598
NC_007643	*Rhodospirillum rubrum* ATCC 11170	4352825
NC_007109	*Rickettsia felis* URRWXCal2	1485148
NC_006142	*Rickettsia typhi* str. Wilmington	1111496
NC_006511	*Salmonella enterica* subsp. *enterica* serovar Paratyphi A str. ATCC	4585229
NC_008750	*Shewanella* sp. W3-18-1	4708380
NC_004741	*Shigella flexneri* 2a str. 2457T	4599354
NC_007384	*Shigella sonnei* Ss046	4825265
NC_009009	*Streptococcus sanguinis* SK36	2388435
NC_007181	*Sulfolobus acidocaldarius* DSM 639	2225959
NC_006177	*Symbiobacterium thermophilum* IAM 14863	3566135
NC_007333	*Thermobifida fusca* YX	3642249
NC_006624	*Thermococcus kodakarensis* KOD1	2088737
NC_002578	*Thermoplasma acidophilum* DSM 1728	1564906
NC_002689	*Thermoplasma volcanium* GSS1	1584804
NC_008312	*Trichodesmium erythraeum* IMS101	7750108
NC_004572	*Tropheryma whipplei* str. Twist	927303
NC_002505	*Vibrio cholerae* O1 biovar eltor str. N16961	2961149
NC_002488	*Xylella fastidiosa* 9a5c	2679306

ii. Details of the simulated benchmark datasets

Details of datasets at family level

simLC_8kb

Arachne

Taxa	*#ofContig*
Bradyrhizobiaceae	202

Phrap

Taxa	*#ofContig*
Bradyrhizobiaceae	229

simMC_8kb

Arachne

Taxa	*#ofContig*
Bradyrhizobiaceae	201
Xanthomonadaceae	100

Phrap

Taxa	*#ofContig*
Bradyrhizobiaceae	296
Xanthomonadaceae	105

simLC_10reads

Arachne

Taxa	*#ofContig*
Bradyrhizobiaceae	348
Flexibacteraceae	6
Gloeocapsaceae@	5
Xanthomonadaceae	7
Burkholderiaceae	1

Phrap

Taxa	*#ofContig*
Bradyrhizobiaceae	428
Flexibacteraceae	23
Xanthomonadaceae	16
Geobacteraceae	1
Gloeocapsaceae@	7
Methylophilaceae	1
Pelobacteraceae	1
Shewanellaceae	1
Streptococcaceae	1
Moraxellaceae	1
Burkholderiaceae	1
Ferroplasmaceae	1

simMC_10reads

Arachne

Taxa	*#ofContig*
Bradyrhizobiaceae	1053
Xanthomonadaceae	311
Moraxellaceae	1
Pelobacteraceae	1
Rhodospirillaceae	3
Gloeocapsaceae@	3

Phrap

Taxa	*#ofContig*
Bradyrhizobiaceae	1398
Xanthomonadaceae	555
Nitrosomonadaceae	1
Rhodospirillaceae	9
Alteromonadaceae	1
Streptococcaceae	1
Shewanellaceae	2
Burkholderiaceae	2
Moraxellaceae	2
Pseudomonadaceae	1
Gloeocapsaceae@	7
Pelobacteraceae	1

Details of datasets at order level

simLC_8kb

Arachne

Taxa	*#ofContig*
Rhizobiales	202

Phrap

Taxa	*#ofContig*
Rhizobiales	229

simMC_8kb

Arachne

Taxa	*#ofContig*
Rhizobiales	201
Xanthomonadales	100

Phrap

Taxa	*#ofContig*
Rhizobiales	296
Xanthomonadales	105

simLC_10reads

Arachne

Taxa	*#ofContig*
Rhizobiales	348
Sphingobacteriales	6
Chroococcales	5
Xanthomonadales	7
Burkholderiales	1

Phrap

Taxa	*#ofContig*
Rhizobiales	428
Sphingobacteriales	23
Xanthomonadales	16
Desulfuromonadales	2
Chroococcales	7
Methylophilales	1
Alteromonadales	1
Lactobacillales	1
Pseudomonadales	1
Burkholderiales	1
Thermoplasmatales	1

simMC_10reads

Arachne

Taxa	*#ofContig*
Rhizobiales	1053
Xanthomonadales	311
Pseudomonadales	1
Desulfuromonadales	1
Rhodospirillales	3
Chroococcales	3

Phrap

Taxa	*#ofContig*
Rhizobiales	1398
Xanthomonadales	555
Nitrosomonadales	1
Rhodospirillales	9
Alteromonadales	3
Lactobacillales	1
Burkholderiales	2
Pseudomonadales	3
Chroococcales	7
Desulfuromonadales	1

Details of datasets at class level

simLC_8kb

Arachne

Taxa	*#ofContig*
Alphaproteobacteria	202

Phrap

Taxa	*#ofContig*
Alphaproteobacteria	229

simMC_8kb

Arachne

Taxa	*#ofContig*
Alphaproteobacteria	201
Gammaproteobacteria	100

Phrap

Taxa	*#ofContig*
Alphaproteobacteria	296
Gammaproteobacteria	105

simLC_10reads

Arachne

Taxa	*#ofContig*
Alphaproteobacteria	348
Sphingobacteria	6
Cyanobacteria	5
Gammaproteobacteria	7
Betaproteobacteria	1

Phrap

Taxa	*#ofContig*
Alphaproteobacteria	428
Sphingobacteria	23
Gammaproteobacteria	18
Deltaproteobacteria	2
Cyanobacteria	7
Betaproteobacteria	2
Bacilli	1
Thermoplasmata	1

simMC_10reads

Arachne

Taxa	*#ofContig*
Alphaproteobacteria	1056
Gammaproteobacteria	312
Deltaproteobacteria	1
Cyanobacteria	3

Phrap

Taxa	*#ofContig*
Alphaproteobacteria	1407
Gammaproteobacteria	561
Betaproteobacteria	3
Bacilli	1
Cyanobacteria	7
Deltaproteobacteria	1

2. Semi-Supervised Clustering Algorithms

COP K-Means [1]

COP K-Means stands for COnstraint-Partitioning K-Means. It uses labelled instances as constraints to restrict the K-Means clustering process. All pairs of labelled instances are marked as either ‘must-link’ or ‘cannot-link’, which are the constraints for two instances having the same or different labels respectively. During the clustering process, a feasible partition will be produced to satisfy all these constraints. Since a different initialization of the K-centres will result in a different solution, we take the best solution in 100 runs with random K-centre initialization.

Seeded K-Means [2]

Instead of using the labelled instances as constraints to restrict the clustering process, Seeded K-Means uses them to initialise the K-centres. When there is more than one labelled instance in a class, the average is taken to be the initial centre. Since, the initialisation of this algorithm is restricted by the labelled data, no multiple initialisations are required.

Constrained K-Means [2]

This algorithm is similar to the combination of COP-KMeans and Seeded-KMeans. It uses the labelled instances to initialise the K-centres as well as restrict the clustering process when finding the feasible partitioning.

Multi-class Transductive Support Vector Machine (TSVM)

Support Vector Machine (SVM) is a supervised two-class classification algorithm. Transductive Support Vector Machine (TSVM) is a semi-supervised two-class learning algorithm. SVM finds a hyper-plane to be placed at the maximum margin between the two labelled classes. Additionally, in TSVM, the hyper-plane is adjusted towards boundary of low density unlabelled instances. We used the available program: SVM-Light (http://svmlight.joachims.org) in this paper. SVM-Light implements the TSVM as described by Joachims [3].

Clustering sequence fragments from several genomes is a multi-class problem. We employ the multi-class architecture proposed by Bruzzone et al. [4] to implement the multi-class TSVM. The architecture diagram is shown below:

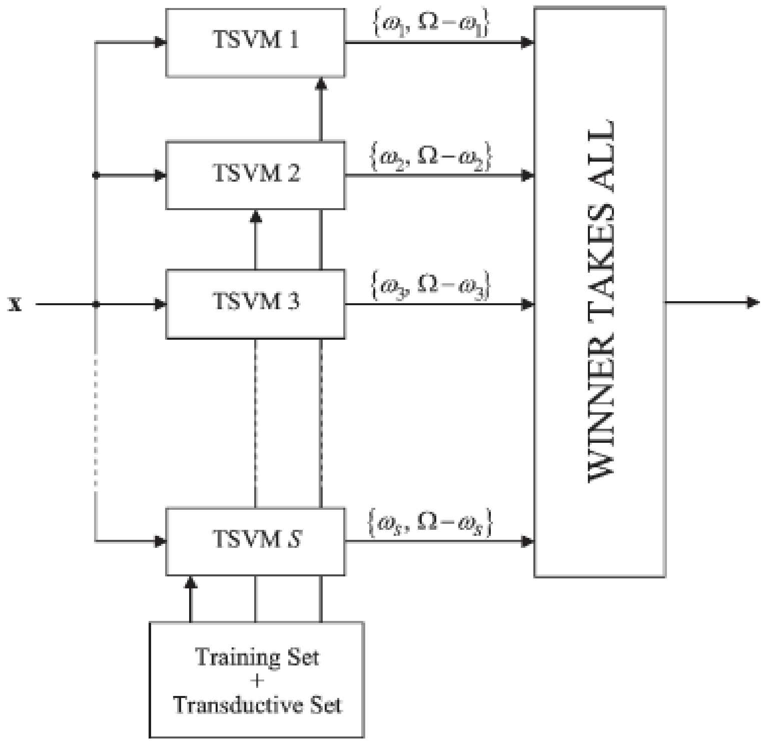

Figure 1: The multi-class TSVM architecture

3. Clustering Performance Measures

F-measure calculation for clustering evaluation

F-measure was introduced by van Rijsbergen [5] in the field of information retrieval to evaluate the effectiveness of a retrieval system. It has become a traditional clustering evaluation measure when the actual class label can be achieved for evaluation.

The calculation of F-measure is clearer to be presented with a contingency table:

	B_1	B_i	…	B_m	Sum	Recall	F-measure
A_1	n_{11}	n_{1i}	...	n_{1m}	$n_{1.}$	R_1	F_1
A_i	n_{i1}	n_{ii}	...	n_{im}	$n_{i.}$	R_i	F_i
⋮	⋮	⋮		⋮	⋮	⋮	⋮
A_m	n_{m1}	n_{mi}	...	n_{mm}	$n_{m.}$	R_m	F_m
Sum	$n_{.1}$	$n_{.i}$	...	$n_{.m}$	N		
Precision	P_1	P_i	...	P_m			

Where

- m is the number of class
- $A_1, A_i, \ldots, A_m$ are the actual classes
- $B_1, B_i, \ldots, B_m$ are the predicted classes
- n_{ii} is the number of correctly predicted samples (Number of B_i predict as A_i)
- $n_{i.} = \sum_j^m n_{ij}$ is the total number of samples are in the actual class A_i
- $n_{.i} = \sum_j^m n_{ji}$ is the total number of samples are in the predicted class B_i
- $N = \sum_j^m n_{j.} = \sum_j^m n_{.j}$ is the total number of samples in the dataset
- $R_i = \frac{n_{ii}}{n_{i.}}$ is the Recall value for A_i
- $P_i = \frac{n_{ii}}{n_{.i}}$ is the Precision value for B_i
- $F_i = \frac{2 \times P_i \times R_i}{P_i + R_i}$ is the F-measure for class A_i

Finally, the weighted F-measure for the clustering result is calculated from:

$$F = \sum_j^m \frac{n_{j.}}{N} F_j$$

Adjusted Rand Index calculation for clustering evaluation

The Rand Index was proposed by [6] to evaluate the clustering quality when the correct labels can be used during the evaluation. Later, the Adjusted Rand Index was introduced by [7] to correct the non-zero expected value of Rand Index even with randomly distributed clusters. The Adjusted Rand Index has become a popular clustering evaluation measure when the actual class label can be achieved for evaluation.

The following contingency table is used to demonstrate the calculation of Adjusted Rand Index:

	B_1	B_j	…	B_k	Sum
A_1	n_{11}	n_{1j}	...	n_{1k}	$n_{1.}$
A_i	n_{i1}	n_{ij}	...	n_{ik}	$n_{i.}$
$\vdots$	$\vdots$	$\vdots$		$\vdots$	$\vdots$
A_m	n_{m1}	n_{mj}	...	n_{mk}	$n_{m.}$
Sum	$n_{.1}$	$n_{.j}$	...	$n_{.k}$	N

Where

- m is the number of actual class
- k is the number of predicted classes
- $A_1, A_i, \ldots, A_m$ are the actual classes
- $B_1, B_j, \ldots, B_k$ are the predicted classes
- $n_{i.} = \sum_{j}^{k} n_{ij}$ is the total number of samples are in the actual class A_i
- $n_{.j} = \sum_{i}^{m} n_{ij}$ is the total number of samples are in the predicted class B_j
- $N = \sum_{i}^{m} n_{i.} = \sum_{j}^{k} n_{.j}$ is the total number of samples in the dataset

The Adjusted Rand Index is calculated using the following equation:

$$\frac{\sum_{i,j}\binom{n_{ij}}{2} - \sum_{i}\binom{n_{i.}}{2}\sum_{j}\binom{n_{.j}}{2} \Big/ \binom{N}{2}}{\frac{1}{2}\left[\sum_{i}\binom{n_{i.}}{2} + \sum_{j}\binom{n_{.j}}{2}\right] - \sum_{i}\binom{n_{i.}}{2}\sum_{j}\binom{n_{.j}}{2} \Big/ \binom{N}{2}}$$

4. Binning Algorithms

Since the binning results of these algorithms presented in this paper were generated by Mavromatis et. al. [8], the detail explanations and settings of the algorithms can be found in the supplementary material of that paper. For the completeness of this paper, these algorithms are briefly described here.

BLAST

This method is a sequence base binning method. It used fgenesb to predict the genes in the metagenomic sequences against the NCBI protein sequences. Then it assigned the sequences to taxonomic classes according to the distribution of BLAST hits of the predicted genes against the reference databases.

k-mer [9]

This is a composition-base binning method. It assigns the sequence fragments to the taxonomic family of the best matching isolate bin by calculating the oligonucleotide frequencies of the sequences and compared to a reference set of finished genomes.

PhyloPythia [10]

PhyloPythia is also a composition-base binning method. It assigned the sequence fragments to phylogenetic clades by comparing the oligonucleotide composition patterns between the metagenomic sequences and a reference set of genomes. There are two models can be built: generic model and species-specific model. The creation of the generic model uses the sequence patterns of a reference set of isolate genomes and the sample-specific model additionally includes about 100 kb of marker-gene labelled sequence fragments from each of the dominant population of the sample. There is one p-value setting (confidence setting) which is determined by the distance of the sample sequences from the hyperplane of the classifier. For example, a higher p-vlaue (eg. p:0.85) means that only samples having a large enough distance to the hyperplane of a specific clade will be assigned to the clade.

5. Result evaluation procedures

In the following, the exact data extraction and evaluation procedure is described and the URLs of the required files are listed at the end of this section.

1. Use the ContigReads.txt (A) and the FASTA format contig files (B) to obtain contigs that are >= 8 kb (in which the wildcard bases 'N' does not take into account for the actual sequence length) or are >= 10 reads.
2. Use the contig_assignment.txt (C) to find out all taxonomic levels (as the true class) for each eligible contigs ranging from domain down to strain level. Let us call this the ActualContigTaxa file. E.g. In the case for "simLC, >= 10 reads, phrap", there are 482 contigs in the ActualContigTaxa file. In this dataset, at the order level, there are 11 different actual taxa (as shown in Table 8).

Table 8: The unique taxa and number of contigs at order level for dataset "simLC, >= 10 reads, phrap"

ID	Taxonomic name	Number of contigs
A1	Rhizobiales	428
A2	Sphingobacteriales	23
A3	Xanthomonadales	16
A4	Desulfuromonadales	2
A5	Chroococcales	7
A6	Methylophilales	1
A7	Alteromonadales	1
A8	Lactobacillales	1
A9	Pseudomonadales	1
A10	Burkholderiales	1
A11	Thermoplasmatales	1

3. We use the predicted taxa of contigs (D) for different binning methods to match the contig_ID in ActualContigTaxa file. Additionally, we use the NCBI database to determine the taxonomy of the predicted taxa names. For example, a contig is predicted to be of taxon Rhizobiales, using NCBI database it is known to be at the order level, as well as all its higher rank (kingdom, phylum, class) taxonomic names. In the same example as above, 478 out of 482 contigs are assigned a predicted taxon by the "gen PhyloPythia p=0.5". However,

some contigs can be predicted to be of a taxon that is not in the list of actual taxa.

4. The contigs that were predicted at or below the specified taxonomic level of comparison are identified. The number of contigs for each taxon is counted and placed into a contingency table with the actual taxa as the row and the predicted taxa as the column names. E.g. in the above example, out of 482 contigs in SimLC dataset that has >= 10 reads, there were 369 contigs predicted at the order level or below. However, within these 369 contigs, 1 contig has the order taxonomic label that is not in the 11 actual taxa so it was treated as unassigned. Therefore, **only 368 contigs were binned**. The exact contig count for each actual taxa and for each predicted taxa are shown in the following contingency table:

	**P1**	**P2**	**P3**	**P4**	**P5**	**P6**	**P7**	**P8**	**P9**	**P10**	**P11**	**UnAsgn**	**Sum**	**Sp**	**Sn**
**A1**	350	0	0	0	0	0	0	0	0	0	0	78	428	1	0.8177
**A2**	0	0	0	0	0	0	0	0	0	0	0	23	23	1	0
**A3**	0	0	15	0	0	0	0	0	0	0	0	1	16	0.9978	0.9375
**A4**	0	0	0	1	0	0	0	0	1	0	0	0	2	1	0.5
**A5**	0	0	0	0	0	0	0	0	0	0	0	7	7	1	0
**A6**	0	0	0	0	0	0	0	0	0	0	0	1	1	1	0
**A7**	0	0	0	0	0	0	0	0	0	0	0	1	1	1	0
**A8**	0	0	0	0	0	0	0	0	0	0	0	1	1	1	0
**A9**	0	0	0	0	0	0	0	0	0	0	0	1	1	0.9979	0
**A10**	0	0	1	0	0	0	0	0	0	0	0	0	1	1	0
**A11**	0	0	0	0	0	0	0	0	0	0	0	1	1	1	0
												Total	482		

where:
Ax represents an actual taxon in dataset. The corresponding taxon name can be found in Table 8.
Px has the same name as the corresponding Ax but representing the predicted taxon.
UnAsgn column is the number of contigs that are not assigned by this method at the given taxa level.
Sp is the specificity for the corresponding class
Sn is the sensitivity for the corresponding class

Since only P1, P3, P4 and P9 contain predicted contigs, so **the number of bins is 4**.

5. Finally, the weighted specificity (wSp) and sensitivity (wSn) can be calculated by:

$$wSp = \frac{428}{482} \times 1 + \frac{23}{482} \times 1 + \frac{16}{482} \times 0.9978 + \cdots$$

wSp = 0.9999

$$wSn = \frac{428}{482} \times 0.8177 + \frac{23}{482} \times 0 + \frac{16}{482} \times 0.9375 + \cdots$$

wSn = 0.7593

The following shows the links in FAMeS to download the mentioned files:
(A): ContigReads.txt.gz:
ftp://ftp.jgi-psf.org/pub/JGI_data/kmavromm/fames/Assemblies/ContigReads.txt.gz
(B): Different files for different datasets were from the 'Assemblers' section in:
http://fames.jgi-psf.org/Retrieve_data.html
(C): contig_assignment.txt.gz:
ftp://ftp.jgi-psf.org/pub/JGI_data/kmavromm/fames/Assemblies/contig_assignment.txt.gz
(D): Predicted taxonomic results were from the 'Binning Methods' section in:
http://fames.jgi-psf.org/Retrieve_data.html

6. Result comparisons of different methods at different taxonomic level for the simulated benchmark datasets

Each of the following tables compares the binning methods for different datasets of complexity (simLC & simMC), different assembler (Arachne & Phrap), different types of datasets (>=8 kb & >= 10 reads) at different taxonomic levels (family, order and class).

The following short forms are used in the tables:

- Total#Contigs: Total number of contigs in the dataset
- %ofBinContigs: The percentage of contigs binned
- #PredNotInAct: The number of contigs predicted as a taxon that is not present in the dataset, which are treated as the un-binned contigs
- wSp: Weighted specificity
- wSn: Weighted sensitivity.

The method with the highest values of wSp and wSn are shown in bold font in the tables.

i. Family level, >=8 kb, simLC

Assembler	Method	Bins	BinnedContigs	Total#Contigs	%ofBinContigs	#PredNotInAct	aSp	wSp	aSn	wSn
Arachne	kmer (7mer)	0	0	202	0	85	-	-	0.000	0.000
Arachne	kmer (8mer)	0	0	202	0	148	-	-	0.000	0.000
Arachne	BLAST distr 1	0	0	202	0	0	-	-	0.000	0.000
Arachne	BLAST distr 2	0	0	202	0	0	-	-	0.000	0.000
Arachne	S-GSOM (CP=55%)	1	141	202	69.8	0	1.000	1.000	0.698	0.698
Arachne	gen PhyloPythia (p:0.85)	0	0	202	0	0	-	-	0.000	0.000
Arachne	ssp PhyloPythia (p:0.85)	1	114	202	56.44	0	1.000	1.000	0.564	0.564
Arachne	S-GSOM (CP=75%)	1	180	202	89.11	0	1.000	1.000	0.891	0.891
Arachne	gen PhyloPythia (p:0.5)	0	0	202	0	0	-	-	0.000	0.000
**Arachne**	**ssp PhyloPythia (p:0.5)**	**1**	**192**	**202**	**95.05**	**0**	**1.000**	**1.000**	**0.950**	**0.950**
Phrap	kmer (7mer)	0	0	229	0	129	-	-	0.000	0.000
Phrap	kmer (8mer)	0	0	229	0	154	-	-	0.000	0.000
Phrap	BLAST distr 1	0	0	229	0	0	-	-	0.000	0.000
Phrap	BLAST distr 2	0	0	229	0	0	-	-	0.000	0.000
Phrap	S-GSOM (CP=55%)	1	157	229	68.56	0	1.000	1.000	0.686	0.686
Phrap	gen PhyloPythia (p:0.85)	0	0	229	0	0	-	-	0.000	0.000
Phrap	ssp PhyloPythia (p:0.85)	1	178	229	77.73	0	1.000	1.000	0.777	0.777
Phrap	S-GSOM (CP=75%)	1	204	229	89.08	0	1.000	1.000	0.891	0.891
Phrap	gen PhyloPythia (p:0.5)	0	0	229	0	0	-	-	0.000	0.000
**Phrap**	**ssp PhyloPythia (p:0.5)**	**1**	**216**	**229**	**94.32**	**0**	**1.000**	**1.000**	**0.943**	**0.943**

ii. Family level, >=8 kb, simMC

Assembler	Method	Bins	BinnedContigs	Total#Contigs	%ofBinContigs	#PredNotInAct	aSp	wSp	aSn	wSn
Arachne	kmer (7mer)	0	0	301	0	47	-	-	0.000	0.000
Arachne	kmer (8mer)	0	0	301	0	191	-	-	0.000	0.000
Arachne	BLAST distr 1	0	0	301	0	0	-	-	0.000	0.000
Arachne	BLAST distr 2	0	0	301	0	0	-	-	0.000	0.000
Arachne	S-GSOM (CP=55%)	2	220	301	73.09	0	1.000	1.000	0.618	0.731
Arachne	gen PhyloPythia (p:0.85)	0	0	301	0	0	-	-	0.000	0.000
Arachne	ssp PhyloPythia (p:0.85)	2	18	301	5.98	0	1.000	1.000	0.067	0.060
**Arachne**	**S-GSOM (CP=75%)**	**2**	**279**	**301**	**92.69**	**0**	**1.000**	**1.000**	**0.890**	**0.927**
Arachne	gen PhyloPythia (p:0.5)	0	0	301	0	0	-	-	0.000	0.000
Arachne	ssp PhyloPythia (p:0.5)	2	143	301	47.51	0	1.000	1.000	0.401	0.475
Phrap	kmer (7mer)	0	0	401	0	84	-	-	0.000	0.000
Phrap	kmer (8mer)	0	0	401	0	271	-	-	0.000	0.000
Phrap	BLAST distr 1	0	0	401	0	0	-	-	0.000	0.000
Phrap	BLAST distr 2	0	0	401	0	0	-	-	0.000	0.000
Phrap	S-GSOM (CP=55%)	2	318	401	79.3	0	1.000	1.000	0.611	0.793
Phrap	gen PhyloPythia (p:0.85)	0	0	401	0	0	-	-	0.000	0.000
Phrap	ssp PhyloPythia (p:0.85)	2	73	401	18.2	0	1.000	1.000	0.323	0.182
**Phrap**	**S-GSOM (CP=75%)**	**2**	**367**	**401**	**91.52**	**0**	**1.000**	**1.000**	**0.841**	**0.915**
Phrap	gen PhyloPythia (p:0.5)	0	0	401	0	0	-	-	0.000	0.000
Phrap	ssp PhyloPythia (p:0.5)	2	87	401	21.7	0	1.000	1.000	0.371	0.217

iii. Family level, >=10 reads, simLC

Assembler	Method	Bins	BinnedContigs	Total#Contigs	%ofBinContigs	#PredNotInAct	aSp	wSp	aSn	wSn
Arachne	kmer (7mer)	0	0	367	0	168	-	-	0.000	0.000
Arachne	kmer (8mer)	0	0	367	0	305	-	-	0.000	0.000
Arachne	BLAST distr 1	0	0	367	0	0	-	-	0.000	0.000
Arachne	BLAST distr 2	0	0	367	0	0	-	-	0.000	0.000
Arachne	S-GSOM (CP=55%)	3	295	367	80.38	0	0.999	1.000	0.168	0.798
Arachne	gen PhyloPythia (p:0.85)	0	0	367	0	0	-	-	0.000	0.000
Arachne	ssp PhyloPythia (p:0.85)	1	116	367	31.61	0	1.000	1.000	0.067	0.316
**Arachne**	**S-GSOM (CP=75%)**	**3**	**343**	**367**	**93.46**	**0**	**0.988**	**0.950**	**0.195**	**0.926**
Arachne	gen PhyloPythia (p:0.5)	0	0	367	0	0	-	-	0.000	0.000
Arachne	ssp PhyloPythia (p:0.5)	1	209	367	56.95	0	1.000	1.000	0.120	0.569
Phrap	kmer (7mer)	0	0	482	0	162	-	-	0.000	0.000
Phrap	kmer (8mer)	2	6	482	1.24	292	0.999	1.000	0.000	0.000
Phrap	BLAST distr 1	0	0	482	0	0	-	-	0.000	0.000
Phrap	BLAST distr 2	0	0	482	0	0	-	-	0.000	0.000
Phrap	S-GSOM (CP=55%)	9	381	482	79.05	9	0.995	1.000	0.084	0.728
Phrap	gen PhyloPythia (p:0.85)	0	0	482	0	0	-	-	0.000	0.000
Phrap	ssp PhyloPythia (p:0.85)	2	199	482	41.29	0	1.000	1.000	0.039	0.411
**Phrap**	**S-GSOM (CP=75%)**	**9**	**443**	**482**	**91.91**	**9**	**0.993**	**1.000**	**0.132**	**0.840**
Phrap	gen PhyloPythia (p:0.5)	0	0	482	0	0	-	-	0.000	0.000
Phrap	ssp PhyloPythia (p:0.5)	3	300	482	62.24	0	1.000	1.000	0.062	0.620

iv. Family level, >=10 reads, simMC

Assembler	Method	Bins	BinnedContigs	Total#Contigs	%ofBinContigs	#PredNotInAct	aSp	wSp	aSn	wSn
Arachne	kmer (7mer)	1	2	1372	0.15	132	1.000	1.000	0.000	0.000
Arachne	kmer (8mer)	0	0	1372	0	1241	-	-	0.000	0.000
Arachne	BLAST distr 1	0	0	1372	0	0	-	-	0.000	0.000
Arachne	BLAST distr 2	0	0	1372	0	0	-	-	0.000	0.000
Arachne	S-GSOM (CP=55%)	5	1061	1372	77.33	0	0.999	0.998	0.454	0.768
Arachne	gen PhyloPythia (p:0.85)	0	0	1372	0	0	-	-	0.000	0.000
Arachne	ssp PhyloPythia (p:0.85)	2	190	1372	13.85	0	1.000	1.000	0.045	0.138
**Arachne**	**S-GSOM (CP=75%)**	**5**	**1253**	**1372**	**91.33**	**0**	**0.995**	**0.983**	**0.539**	**0.897**
Arachne	gen PhyloPythia (p:0.5)	0	0	1372	0	0	-	-	0.000	0.000
Arachne	ssp PhyloPythia (p:0.5)	2	673	1372	49.05	0	1.000	1.000	0.141	0.491
Phrap	kmer (7mer)	1	1	1980	0.05	148	1.000	1.000	0.000	0.000
Phrap	kmer (8mer)	1	53	1980	2.68	1795	0.998	1.000	0.000	0.000
Phrap	BLAST distr 1	0	0	1980	0	0	-	-	0.000	0.000
Phrap	BLAST distr 2	0	0	1980	0	0	-	-	0.000	0.000
Phrap	S-GSOM (CP=55%)	10	1409	1980	71.16	9	0.997	0.995	0.160	0.686
Phrap	gen PhyloPythia (p:0.85)	0	0	1980	0	0	-	-	0.000	0.000
Phrap	ssp PhyloPythia (p:0.85)	2	172	1980	8.69	0	1.000	1.000	0.018	0.087
**Phrap**	**S-GSOM (CP=75%)**	**10**	**1708**	**1980**	**86.26**	**9**	**0.995**	**0.991**	**0.195**	**0.816**
Phrap	gen PhyloPythia (p:0.5)	0	0	1980	0	0	-	-	0.000	0.000
Phrap	ssp PhyloPythia (p:0.5)	2	367	1980	18.54	0	1.000	1.000	0.035	0.185

v. Order level, >=8 kb, simLC

Assembler	Method	Bins	BinnedContigs	Total#Contigs	%ofBinContigs	#PredNotInAct	aSp	wSp	aSn	wSn
Arachne	kmer (7mer)	0	0	202	0	85	-	-	0.000	0.000
Arachne	kmer (8mer)	0	0	202	0	149	-	-	0.000	0.000
Arachne	BLAST distr 1	0	0	202	0	0	-	-	0.000	0.000
Arachne	BLAST distr 2	0	0	202	0	0	-	-	0.000	0.000
Arachne	S-GSOM (CP=55%)	1	141	202	69.8	0	1.000	1.000	0.698	0.698
Arachne	gen PhyloPythia (p:0.85)	1	168	202	83.17	0	1.000	1.000	0.832	0.832
Arachne	ssp PhyloPythia (p:0.85)	1	186	202	92.08	0	1.000	1.000	0.921	0.921
Arachne	S-GSOM (CP=75%)	1	180	202	89.11	0	1.000	1.000	0.891	0.891
**Arachne**	**gen PhyloPythia (p:0.5)**	**1**	**201**	**202**	**99.5**	**0**	**1.000**	**1.000**	**0.995**	**0.995**
**Arachne**	**ssp PhyloPythia (p:0.5)**	**1**	**201**	**202**	**99.5**	**0**	**1.000**	**1.000**	**0.995**	**0.995**
Phrap	kmer (7mer)	0	0	229	0	129	-	-	0.000	0.000
Phrap	kmer (8mer)	0	0	229	0	154	-	-	0.000	0.000
Phrap	BLAST distr 1	0	0	229	0	0	-	-	0.000	0.000
Phrap	BLAST distr 2	0	0	229	0	0	-	-	0.000	0.000
Phrap	S-GSOM (CP=55%)	1	157	229	68.56	0	1.000	1.000	0.686	0.686
Phrap	gen PhyloPythia (p:0.85)	1	185	229	80.79	0	1.000	1.000	0.808	0.808
Phrap	ssp PhyloPythia (p:0.85)	1	205	229	89.52	0	1.000	1.000	0.895	0.895
Phrap	S-GSOM (CP=75%)	1	204	229	89.08	0	1.000	1.000	0.891	0.891
**Phrap**	**gen PhyloPythia (p:0.5)**	**1**	**227**	**229**	**99.13**	**0**	**1.000**	**1.000**	**0.991**	**0.991**
**Phrap**	**ssp PhyloPythia (p:0.5)**	**1**	**227**	**229**	**99.13**	**0**	**1.000**	**1.000**	**0.991**	**0.991**

vi. Order level, >=8 kb, simMC

Assembler	Method	Bins	BinnedContigs	Total#Contigs	%ofBinContigs	#PredNotInAct	aSp	wSp	aSn	wSn
Arachne	kmer (7mer)	0	0	301	0	47	-	-	0.000	0.000
Arachne	kmer (8mer)	0	0	301	0	191	-	-	0.000	0.000
Arachne	BLAST distr 1	0	0	301	0	0	-	-	0.000	0.000
Arachne	BLAST distr 2	0	0	301	0	0	-	-	0.000	0.000
Arachne	S-GSOM (CP=55%)	2	220	301	73.09	0	1.000	1.000	0.618	0.731
Arachne	gen PhyloPythia (p:0.85)	2	242	301	80.4	0	1.000	1.000	0.838	0.804
Arachne	ssp PhyloPythia (p:0.85)	2	242	301	80.4	0	1.000	1.000	0.841	0.804
Arachne	S-GSOM (CP=75%)	2	279	301	92.69	0	1.000	1.000	0.890	0.927
**Arachne**	**gen PhyloPythia (p:0.5)**	**2**	**301**	**301**	**100**	**0**	**1.000**	**1.000**	**1.000**	**1.000**
**Arachne**	**ssp PhyloPythia (p:0.5)**	**2**	**301**	**301**	**100**	**0**	**1.000**	**1.000**	**1.000**	**1.000**
Phrap	kmer (7mer)	0	0	401	0	84	-	-	0.000	0.000
Phrap	kmer (8mer)	0	0	401	0	271	-	-	0.000	0.000
Phrap	BLAST distr 1	0	0	401	0	0	-	-	0.000	0.000
Phrap	BLAST distr 2	0	0	401	0	0	-	-	0.000	0.000
Phrap	S-GSOM (CP=55%)	2	318	401	79.3	0	1.000	1.000	0.611	0.793
Phrap	gen PhyloPythia (p:0.85)	2	301	401	75.06	0	1.000	1.000	0.803	0.751
Phrap	ssp PhyloPythia (p:0.85)	2	295	401	73.57	0	1.000	1.000	0.787	0.736
Phrap	S-GSOM (CP=75%)	2	367	401	91.52	0	1.000	1.000	0.841	0.915
**Phrap**	**gen PhyloPythia (p:0.5)**	**2**	**399**	**401**	**99.5**	**1**	**1.000**	**1.000**	**0.997**	**0.995**
**Phrap**	**ssp PhyloPythia (p:0.5)**	**2**	**399**	**401**	**99.5**	**1**	**1.000**	**1.000**	**0.997**	**0.995**

vii. Order level, >=10 reads, simLC

Assembler	Method	Bins	BinnedContigs	Total#Contigs	%ofBinContigs	#PredNotInAct	aSp	wSp	aSn	wSn
Arachne	kmer (7mer)	0	0	367	0	168	-	-	0.000	0.000
Arachne	kmer (8mer)	0	0	367	0	312	-	-	0.000	0.000
Arachne	BLAST distr 1	0	0	367	0	0	-	-	0.000	0.000
Arachne	BLAST distr 2	0	0	367	0	0	-	-	0.000	0.000
Arachne	S-GSOM (CP=55%)	3	295	367	80.38	0	0.999	1.000	0.168	0.798
Arachne	gen PhyloPythia (p:0.85)	2	214	367	58.31	0	1.000	1.000	0.207	0.583
Arachne	ssp PhyloPythia (p:0.85)	2	236	367	64.31	0	0.999	1.000	0.218	0.638
**Arachne**	**S-GSOM (CP=75%)**	**3**	**343**	**367**	**93.46**	**0**	**0.988**	**0.950**	**0.195**	**0.926**
Arachne	gen PhyloPythia (p:0.5)	2	292	367	79.56	0	1.000	1.000	0.308	0.796
Arachne	ssp PhyloPythia (p:0.5)	2	296	367	80.65	0	0.998	1.000	0.308	0.798
Phrap	kmer (7mer)	2	3	482	0.62	159	0.999	1.000	0.000	0.000
Phrap	kmer (8mer)	3	17	482	3.53	281	0.997	1.000	0.000	0.000
Phrap	BLAST distr 1	0	0	482	0	0	-	-	0.000	0.000
Phrap	BLAST distr 2	0	0	482	0	1	-	-	0.000	0.000
Phrap	S-GSOM (CP=55%)	8	381	482	79.05	9	0.994	1.000	0.091	0.728
Phrap	gen PhyloPythia (p:0.85)	3	236	482	48.96	0	1.000	1.000	0.105	0.488
Phrap	ssp PhyloPythia (p:0.85)	3	272	482	56.43	0	1.000	1.000	0.112	0.560
**Phrap**	**S-GSOM (CP=75%)**	**8**	**443**	**482**	**91.91**	**9**	**0.993**	**1.000**	**0.144**	**0.840**
Phrap	gen PhyloPythia (p:0.5)	4	368	482	76.35	1	1.000	1.000	0.205	0.759
Phrap	ssp PhyloPythia (p:0.5)	5	387	482	80.29	1	0.999	1.000	0.213	0.797

viii. Order level, >=10 reads, simMC

Assembler	Method	Bins	BinnedContigs	Total#Contigs	%ofBinContigs	#PredNotInAct	aSp	wSp	aSn	wSn
Arachne	kmer (7mer)	1	2	1372	0.15	133	1.000	1.000	0.000	0.000
Arachne	kmer (8mer)	0	0	1372	0	1241	-	-	0.000	0.000
Arachne	BLAST distr 1	0	0	1372	0	0	-	-	0.000	0.000
Arachne	BLAST distr 2	0	0	1372	0	1	-	-	0.000	0.000
Arachne	S-GSOM (CP=55%)	5	1061	1372	77.33	0	0.999	0.998	0.454	0.768
Arachne	gen PhyloPythia (p:0.85)	3	562	1372	40.96	0	1.000	1.000	0.156	0.409
Arachne	ssp PhyloPythia (p:0.85)	3	657	1372	47.89	0	1.000	1.000	0.173	0.478
**Arachne**	**S-GSOM (CP=75%)**	**5**	**1253**	**1372**	**91.33**	**0**	**0.995**	**0.983**	**0.539**	**0.897**
Arachne	gen PhyloPythia (p:0.5)	4	1036	1372	75.51	6	1.000	1.000	0.417	0.753
Arachne	ssp PhyloPythia (p:0.5)	4	1102	1372	80.32	4	1.000	1.000	0.428	0.802
Phrap	kmer (7mer)	1	1	1980	0.05	163	1.000	1.000	0.000	0.000
Phrap	kmer (8mer)	2	391	1980	19.75	1457	0.980	1.000	0.000	0.000
Phrap	BLAST distr 1	0	0	1980	0	2	-	-	0.000	0.000
Phrap	BLAST distr 2	0	0	1980	0	3	-	-	0.000	0.000
Phrap	S-GSOM (CP=55%)	8	1409	1980	71.16	9	0.997	0.995	0.175	0.686
Phrap	gen PhyloPythia (p:0.85)	3	799	1980	40.35	1	1.000	1.000	0.101	0.404
Phrap	ssp PhyloPythia (p:0.85)	3	844	1980	42.63	1	1.000	1.000	0.104	0.426
**Phrap**	**S-GSOM (CP=75%)**	**8**	**1708**	**1980**	**86.26**	**9**	**0.994**	**0.991**	**0.218**	**0.816**
Phrap	gen PhyloPythia (p:0.5)	5	1484	1980	74.95	6	1.000	1.000	0.204	0.745
Phrap	ssp PhyloPythia (p:0.5)	5	1524	1980	76.97	4	1.000	1.000	0.208	0.767

ix. Class level, >=8 kb, simLC

Assembler	Method	Bins	BinnedContigs	Total#Contigs	%ofBinContigs	#PredNotInAct	aSp	wSp	aSn	wSn
Arachne	kmer (7mer)	1	82	202	40.59	120	1.000	1.000	0.406	0.406
Arachne	kmer (8mer)	1	27	202	13.37	175	1.000	1.000	0.134	0.134
Arachne	BLAST distr 1	1	149	202	73.76	27	1.000	1.000	0.738	0.738
Arachne	BLAST distr 2	1	158	202	78.22	41	1.000	1.000	0.782	0.782
Arachne	S-GSOM (CP=55%)	1	141	202	69.8	0	1.000	1.000	0.698	0.698
Arachne	gen PhyloPythia (p:0.85)	1	194	202	96.04	0	1.000	1.000	0.960	0.960
Arachne	ssp PhyloPythia (p:0.85)	1	196	202	97.03	0	1.000	1.000	0.970	0.970
Arachne	S-GSOM (CP=75%)	1	180	202	89.11	0	1.000	1.000	0.891	0.891
**Arachne**	**gen PhyloPythia (p:0.5)**	**1**	**201**	**202**	**99.5**	**1**	**1.000**	**1.000**	**0.995**	**0.995**
**Arachne**	**ssp PhyloPythia (p:0.5)**	**1**	**201**	**202**	**99.5**	**1**	**1.000**	**1.000**	**0.995**	**0.995**
Phrap	kmer (7mer)	1	129	229	56.33	100	1.000	1.000	0.563	0.563
Phrap	kmer (8mer)	1	138	229	60.26	91	1.000	1.000	0.603	0.603
Phrap	BLAST distr 1	1	164	229	71.62	29	1.000	1.000	0.716	0.716
Phrap	BLAST distr 2	1	181	229	79.04	41	1.000	1.000	0.790	0.790
Phrap	S-GSOM (CP=55%)	1	157	229	68.56	0	1.000	1.000	0.686	0.686
Phrap	gen PhyloPythia (p:0.85)	1	219	229	95.63	0	1.000	1.000	0.956	0.956
Phrap	ssp PhyloPythia (p:0.85)	1	223	229	97.38	0	1.000	1.000	0.974	0.974
Phrap	S-GSOM (CP=75%)	1	204	229	89.08	0	1.000	1.000	0.891	0.891
**Phrap**	**gen PhyloPythia (p:0.5)**	**1**	**228**	**229**	**99.56**	**1**	**1.000**	**1.000**	**0.996**	**0.996**
**Phrap**	**ssp PhyloPythia (p:0.5)**	**1**	**228**	**229**	**99.56**	**1**	**1.000**	**1.000**	**0.996**	**0.996**

x. Class level, >=8 kb, simMC

Assembler	Method	Bins	BinnedContigs	Total#Contigs	%ofBinContigs	#PredNotInAct	aSp	wSp	aSn	wSn
Arachne	kmer (7mer)	1	47	301	15.61	254	1.000	1.000	0.117	0.156
Arachne	kmer (8mer)	2	74	301	24.58	227	0.945	0.940	0.147	0.196
Arachne	BLAST distr 1	2	255	301	84.72	11	0.935	0.957	0.801	0.761
Arachne	BLAST distr 2	2	274	301	91.03	13	0.910	0.940	0.828	0.791
Arachne	S-GSOM (CP=55%)	2	220	301	73.09	0	1.000	1.000	0.618	0.731
Arachne	gen PhyloPythia (p:0.85)	2	296	301	98.34	0	1.000	1.000	0.983	0.983
Arachne	ssp PhyloPythia (p:0.85)	2	296	301	98.34	0	1.000	1.000	0.985	0.983
Arachne	S-GSOM (CP=75%)	2	279	301	92.69	0	1.000	1.000	0.890	0.927
**Arachne**	**gen PhyloPythia (p:0.5)**	**2**	**301**	**301**	**100**	**0**	**1.000**	**1.000**	**1.000**	**1.000**
**Arachne**	**ssp PhyloPythia (p:0.5)**	**2**	**301**	**301**	**100**	**0**	**1.000**	**1.000**	**1.000**	**1.000**
Phrap	kmer (7mer)	1	81	401	20.2	320	1.000	1.000	0.137	0.202
Phrap	kmer (8mer)	2	39	401	9.73	362	0.943	0.966	0.012	0.017
Phrap	BLAST distr 1	2	324	401	80.8	17	0.943	0.970	0.776	0.723
Phrap	BLAST distr 2	2	360	401	89.78	23	0.922	0.959	0.832	0.783
Phrap	S-GSOM (CP=55%)	2	318	401	79.3	0	1.000	1.000	0.611	0.793
Phrap	gen PhyloPythia (p:0.85)	2	387	401	96.51	0	1.000	1.000	0.973	0.965
Phrap	ssp PhyloPythia (p:0.85)	2	380	401	94.76	0	1.000	1.000	0.955	0.948
Phrap	S-GSOM (CP=75%)	2	367	401	91.52	0	1.000	1.000	0.841	0.915
**Phrap**	**gen PhyloPythia (p:0.5)**	**2**	**400**	**401**	**99.75**	**0**	**1.000**	**1.000**	**0.998**	**0.998**
**Phrap**	**ssp PhyloPythia (p:0.5)**	**2**	**400**	**401**	**99.75**	**0**	**1.000**	**1.000**	**0.998**	**0.998**

xi. Class level, >=10 reads, simLC

Assembler	Method	Bins	BinnedContigs	Total#Contigs	%ofBinContigs	#PredNotInAct	aSp	wSp	aSn	wSn
Arachne	kmer (7mer)	1	93	367	25.34	274	1.000	1.000	0.053	0.253
Arachne	kmer (8mer)	2	54	367	14.71	313	0.986	0.999	0.016	0.076
Arachne	BLAST distr 1	3	281	367	76.57	6	0.971	0.998	0.271	0.621
Arachne	BLAST distr 2	3	327	367	89.1	14	0.959	0.998	0.285	0.689
Arachne	S-GSOM (CP=55%)	3	295	367	80.38	0	0.999	1.000	0.168	0.798
Arachne	gen PhyloPythia (p:0.85)	3	283	367	77.11	1	0.997	1.000	0.272	0.757
Arachne	ssp PhyloPythia (p:0.85)	3	287	367	78.2	1	0.996	1.000	0.273	0.763
**Arachne**	**S-GSOM (CP=75%)**	**3**	**343**	**367**	**93.46**	**0**	**0.988**	**0.950**	**0.195**	**0.926**
**Arachne**	**gen PhyloPythia (p:0.5)**	**3**	**349**	**367**	**95.1**	**1**	**0.985**	**0.950**	**0.563**	**0.926**
Arachne	ssp PhyloPythia (p:0.5)	3	352	367	95.91	1	0.983	0.950	0.562	0.924
Phrap	kmer (7mer)	3	141	482	29.25	341	0.999	1.000	0.040	0.284
Phrap	kmer (8mer)	3	258	482	53.53	224	0.948	0.670	0.063	0.448
Phrap	BLAST distr 1	4	336	482	69.71	18	0.983	0.997	0.166	0.564
Phrap	BLAST distr 2	4	404	482	83.82	29	0.973	0.979	0.184	0.643
Phrap	S-GSOM (CP=55%)	5	381	482	79.05	9	0.992	0.999	0.132	0.730
Phrap	gen PhyloPythia (p:0.85)	3	329	482	68.26	1	0.999	1.000	0.175	0.674
Phrap	ssp PhyloPythia (p:0.85)	3	341	482	70.75	1	0.999	1.000	0.185	0.699
Phrap	S-GSOM (CP=75%)	5	443	482	91.91	9	0.990	0.999	0.204	0.842
Phrap	gen PhyloPythia (p:0.5)	6	446	482	92.53	3	0.997	1.000	0.302	0.900
**Phrap**	**ssp PhyloPythia (p:0.5)**	**7**	**447**	**482**	**92.74**	**3**	**0.997**	**1.000**	**0.307**	**0.902**

xii. Class level, >=10 reads, simMC

Assembler	Method	Bins	BinnedContigs	Total#Contigs	%ofBinContigs	#PredNotInAct	aSp	wSp	aSn	wSn
Arachne	kmer (7mer)	2	125	1372	9.11	1247	0.992	0.975	0.027	0.082
Arachne	kmer (8mer)	2	250	1372	18.22	1122	0.979	0.965	0.047	0.132
Arachne	BLAST distr 1	3	938	1372	68.37	111	0.967	0.964	0.321	0.588
Arachne	BLAST distr 2	3	1102	1372	80.32	155	0.956	0.951	0.364	0.676
Arachne	S-GSOM (CP=55%)	3	1061	1372	77.33	0	0.999	1.000	0.267	0.771
Arachne	gen PhyloPythia (p:0.85)	3	933	1372	68	6	1.000	1.000	0.346	0.679
Arachne	ssp PhyloPythia (p:0.85)	3	980	1372	71.43	6	1.000	1.000	0.357	0.713
Arachne	S-GSOM (CP=75%)	3	1253	1372	91.33	0	0.993	0.985	0.395	0.900
Arachne	gen PhyloPythia (p:0.5)	3	1305	1372	95.12	23	1.000	1.000	0.461	0.950
**Arachne**	**ssp PhyloPythia (p:0.5)**	**3**	**1307**	**1372**	**95.26**	**22**	**1.000**	**1.000**	**0.462**	**0.951**
Phrap	kmer (7mer)	2	142	1980	7.17	1838	1.000	0.999	0.016	0.070
Phrap	kmer (8mer)	3	429	1980	21.67	1551	0.962	0.982	0.002	0.006
Phrap	BLAST distr 1	4	1466	1980	74.04	31	0.971	0.963	0.272	0.606
Phrap	BLAST distr 2	4	1748	1980	88.28	52	0.961	0.947	0.302	0.698
Phrap	S-GSOM (CP=55%)	4	1409	1980	71.16	9	0.998	0.993	0.171	0.706
Phrap	gen PhyloPythia (p:0.85)	4	1337	1980	67.53	2	0.999	1.000	0.226	0.671
Phrap	ssp PhyloPythia (p:0.85)	4	1349	1980	68.13	2	0.999	1.000	0.227	0.677
Phrap	S-GSOM (CP=75%)	4	1708	1980	86.26	9	0.997	0.991	0.254	0.856
Phrap	gen PhyloPythia (p:0.5)	4	1880	1980	94.95	7	0.998	0.999	0.305	0.935
**Phrap**	**ssp PhyloPythia (p:0.5)**	**4**	**1881**	**1980**	**95**	**7**	**0.998**	**0.999**	**0.306**	**0.937**

7. GSOM settings and GSOM maps

GSOM settings in the paper

All results (Including both experiments in comparison of semi-supervised algorithms and simulated metagenomic datasets) were generated using the default GSOM settings. The default GSOM settings are list below:

- Topology Type = Hexagon
- Similarity Measure = Euclidean
- Weight Initialisation Type = 0.5 for all nodes
- Neighbourhood Kernel = Gaussian Kernel
- Initial Learning Rates: Growing Phase = 0.1, First Smoothing Phase = 0.05, Second Smoothing Phase = 0.01
- Training Epochs: Growing Phase = 5, First Smoothing Phase = 50, Second Smoothing Phase = 50

Additionally, the Spread Factors used are:

- SF=0.1 for all the >=8 kb simulated metagenome datasets.
- SF=0.2 for simLC, >=10 read simulated metagenome datasets.
- SF=0.3 for simMC, >=10 read simulated metagenome datasets.
- SF=0.85 for all artificially generated datasets.

GSOM map results for simulated metagenome datasets

The trained GSOM maps for all simulated metagenomic datasets are shown below. Each hexagon represents a single node in the map. These maps are shown in the form of a distance map which visualises the distance of the weight vector of a node to each of its six neighbour nodes. The colour scale shows the exact Euclidean distance value which the colour represents.

Datasets: simLC, ≥8 kb

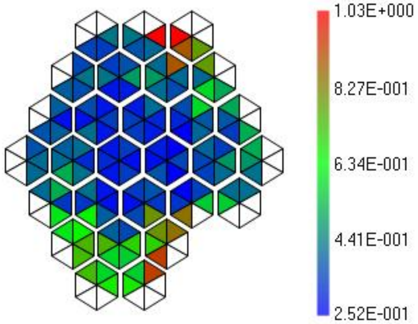

Figure 1: Arachne

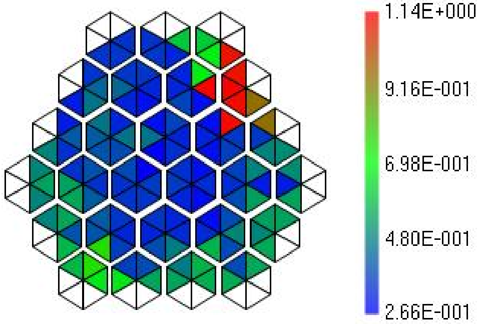

Phrap

Datasets: simMC, ≥8 kb

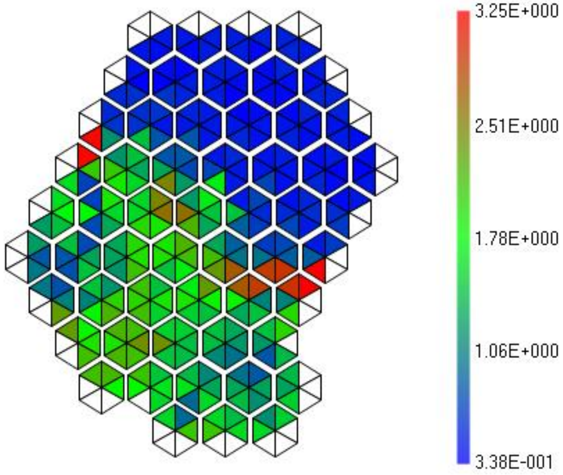

Figure 2: Arachne

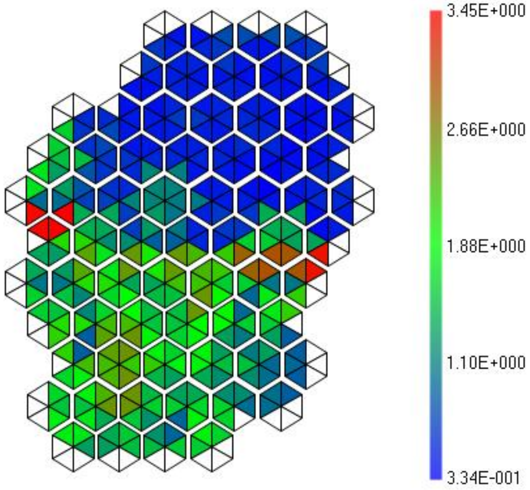

Phrap

Datasets: simLC, ≥10 reads

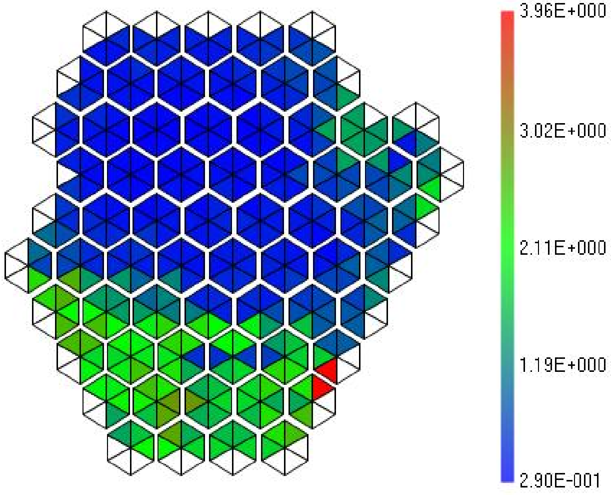

Figure 3: Arachne

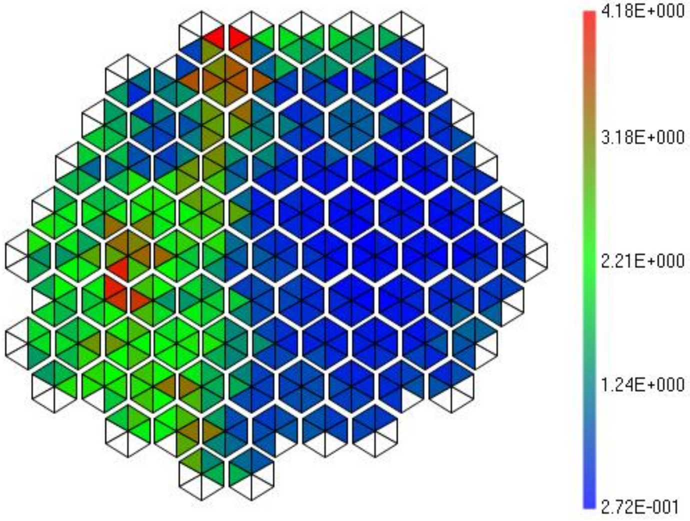

Phrap

Datasets: simMC, ≥10 reads

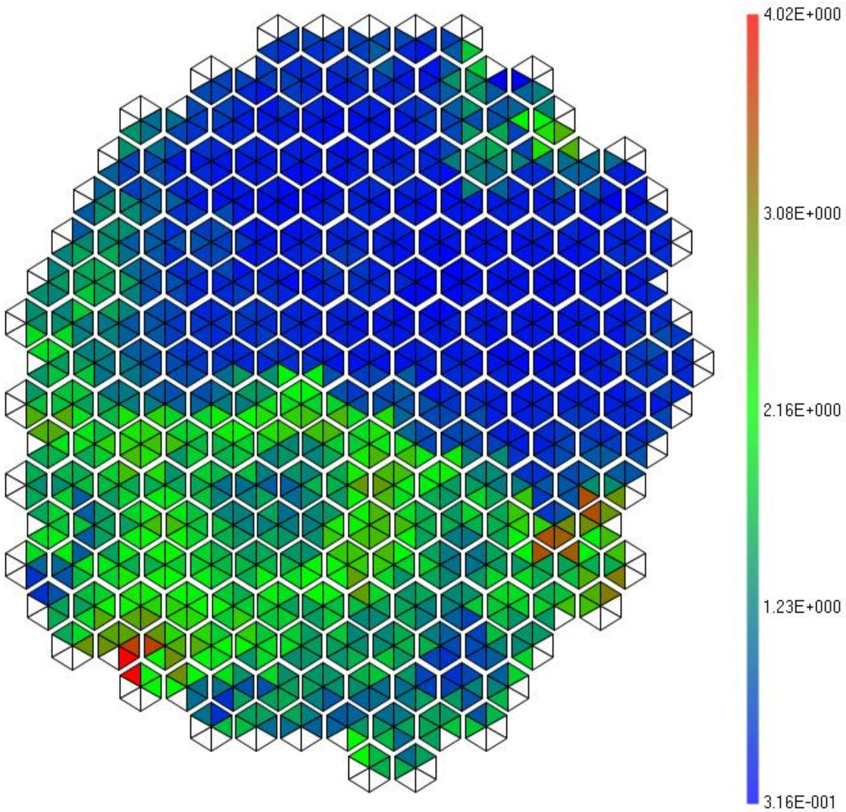

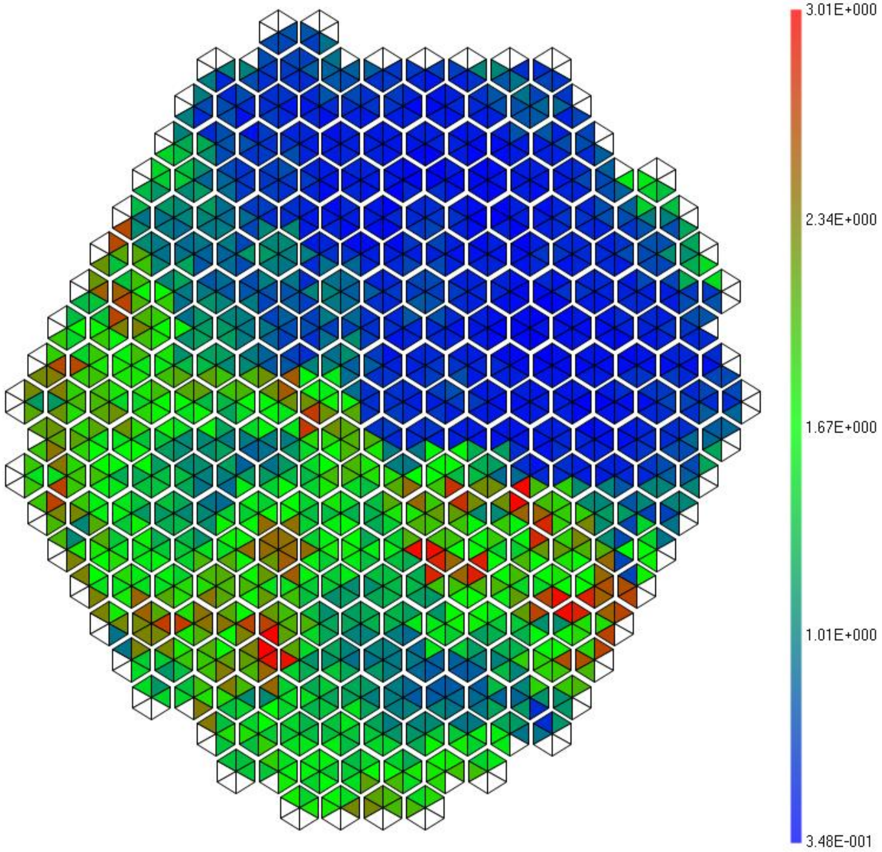

Figure 4: Arachne Phrap

8. Relationship between CP and the percentage of assigned samples

The following graph demonstrates the relationship between CP value and the percentage of assigned samples on the 40 species dataset.

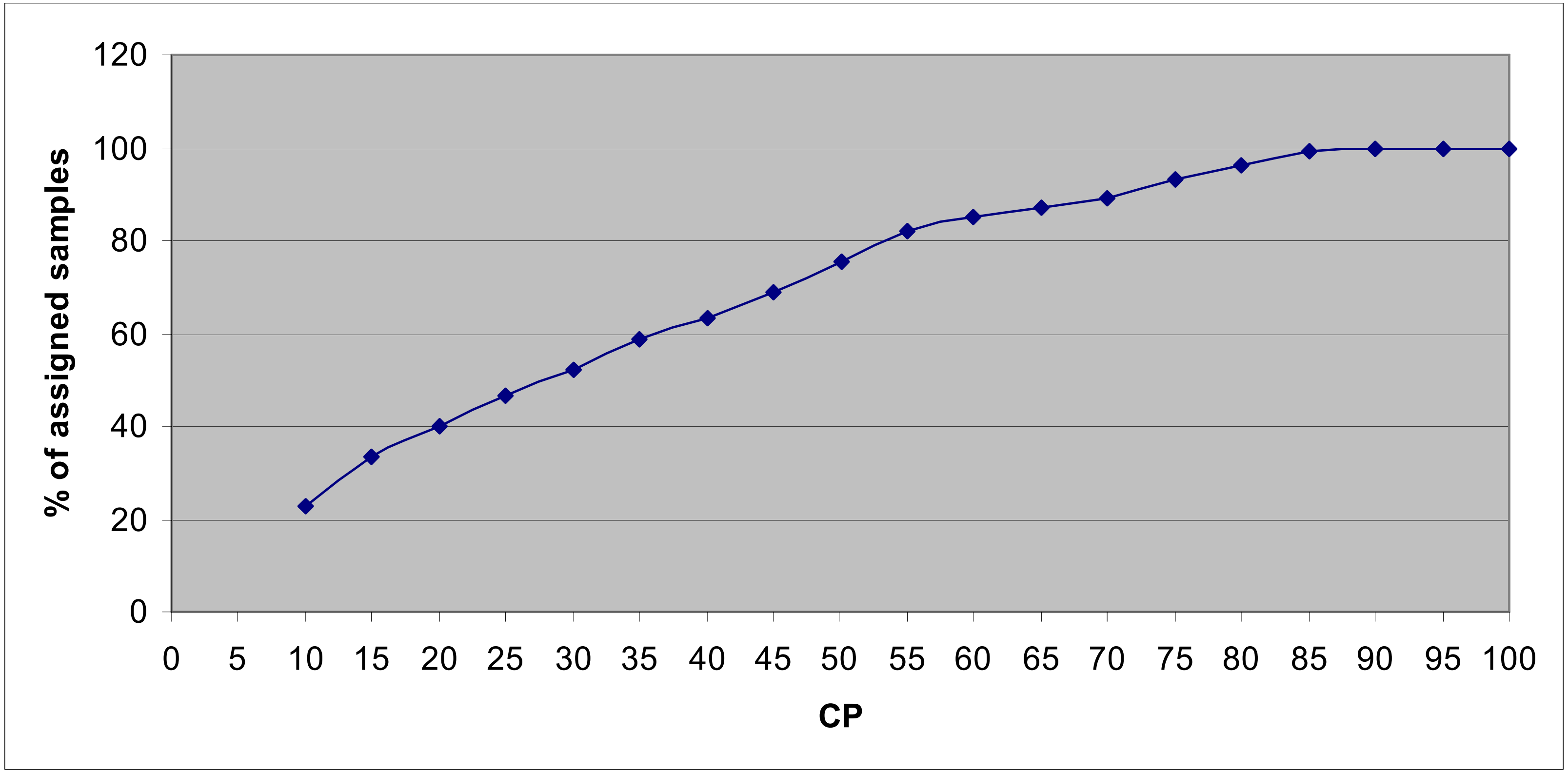

9. References

1. Wagstaff K, Cardie C, Rogers S, Schroedl S: **Constrained K-means Clustering with Background Knowledge**. In: *Proceedings of 18th International Conference on Machine Learning (ICML-01): 2001*; 2001: 577-584.
2. Basu S, Banerjee A, Mooney RJ: **Semi-supervised Clustering by Seeding**. In: *Proceedings of the Nineteenth International Conference on Machine Learning (ICML-2002): July 2002; Sydney, Australia*; 2002: 19-26.
3. Joachims T: **Transductive inference for text classification using support vector machines**. In: *Proceedings of ICML-99, 16th International Conference on Machine Learning: 1999*: Morgan Kaufmann Publishers, San Francisco, US; 1999: 200-209.
4. Bruzzone L, Chi M, Marconcini M: **A Novel Transductive SVM for Semisupervised Classification of Remote-Sensing Images**. *Geoscience and Remote Sensing, IEEE Transactions on* 2006, **44**(11):3363-3373.
5. van Rijsbergen CJ: **Information Retrieval**, 2nd edn. London: Butterworths; 1979.
6. Rand WM: **Objective Criteria for the Evaluation of Clustering Methods**. *Journal of the American Statistical Association* 1971, **66**(336):846-850.
7. Hubert L: **Comparing Partitions**. *Journal of Classification* 1985, **2**:193-218.
8. Mavromatis K, Ivanova N, Barry K, Shapiro H, Goltsman E, McHardy AC, Rigoutsos I, Salamov A, Korzeniewski F, Land M *et al*: **Use of simulated data sets to evaluate the fidelity of metagenomic processing methods**. *Nature Method* 2007, **4**(6):495-500.
9. Sandberg R, Winberg G, Branden C-I, Kaske A, Ernberg I, Coster J: **Capturing Whole-Genome Characteristics in Short Sequences Using a Naive Bayesian Classifier**. *Genome Res* 2001, **11**(8):1404-1409.
10. McHardy AC, Martin HG, Tsirigos A, Hugenholtz P, Rigoutsos I: **Accurate phylogenetic classification of variable-length DNA fragments**. *Nature Methods* 2007, 4(1):63-72.
